# *ARL3* Mutations Cause Joubert Syndrome by Disrupting Ciliary Protein Composition

**DOI:** 10.1016/j.ajhg.2018.08.015

**Published:** 2018-09-27

**Authors:** Sumaya Alkanderi, Elisa Molinari, Ranad Shaheen, Yasmin Elmaghloob, Louise A. Stephen, Veronica Sammut, Simon A. Ramsbottom, Shalabh Srivastava, George Cairns, Noel Edwards, Sarah J. Rice, Nour Ewida, Amal Alhashem, Kathryn White, Colin G. Miles, David H. Steel, Fowzan S. Alkuraya, Shehab Ismail, John A. Sayer

**Affiliations:** 1 Institute of Genetic Medicine, Newcastle University, International Centre for Life, Central Parkway, Newcastle upon Tyne NE1 3BZ, UK; 2 Department of Genetics, King Faisal Specialist Hospital and Research Center, Riyadh 11211, Saudi Arabia; 3 Structural Biology of the Cilia Lab, Beatson Institute for Cancer Research, Switchback Road, Bearsden, Glasgow G61 1BD, UK; 4 Renal Services, Newcastle Hospitals NHS Foundation Trust, Newcastle upon Tyne NE7 7DN, UK; 5 Department of Pediatrics, Prince Sultan Medical Military City, Riyadh 12233, Saudi Arabia; 6 Department of Anatomy and Cell Biology, College of Medicine, Alfaisal University, Riyadh 11533, Saudi Arabia; 7 Electron Microscopy Research Services, Newcastle University, Newcastle upon Tyne NE2 4HH, UK; 8 Sunderland Eye Infirmary, Queen Alexandra Road, Sunderland SR2 9HP, UK; 9 Institute of Cancer Sciences, University of Glasgow, Glasgow G61 1QH, UK

**Keywords:** cilia, Joubert syndrome, trafficking, guanine nucleotide exchange factor, ARL3, ARL13B

## Abstract

Joubert syndrome (JBTS) is a genetically heterogeneous autosomal-recessive neurodevelopmental ciliopathy. We investigated further the underlying genetic etiology of Joubert syndrome by studying two unrelated families in whom JBTS was not associated with pathogenic variants in known JBTS-associated genes. Combined autozygosity mapping of both families highlighted a candidate locus on chromosome 10 (chr10: 101569997–109106128, UCSC Genome Browser hg 19), and exome sequencing revealed two missense variants in *ARL3* within the candidate locus. The encoded protein, ADP ribosylation factor-like GTPase 3 (ARL3), is a small GTP-binding protein that is involved in directing lipid-modified proteins into the cilium in a GTP-dependent manner. Both missense variants replace the highly conserved Arg149 residue, which we show to be necessary for the interaction with its guanine nucleotide exchange factor ARL13B, such that the mutant protein is associated with reduced INPP5E and NPHP3 localization in cilia. We propose that ARL3 provides a potential hub in the network of proteins implicated in ciliopathies, whereby perturbation of ARL3 leads to the mislocalization of multiple ciliary proteins as a result of abnormal displacement of lipidated protein cargo.

## Main Text

Mutations in genes that are involved in the structure or function of the primary cilium give rise to a range of disorders known as ciliopathies.^1^ These are typically multi-system disorders, as seen in the archetypal ciliopathy Joubert syndrome (JBTS), which is characterized clinically by brain malformations that result in developmental delay, oculomotor apraxia, and hypotonia.^2^ In addition to the neurodevelopmental phenotype, retinal and renal diseases are often associated with JBTS.^3^ Now more than 35 genes are known to cause JBTS when mutated in an autosomal-recessive or X-linked manner^4^, ^5^, ^6^, ^7^ (also see GeneReviews in Web Resources). Genetic approaches have moved from traditional linkage studies and homozygosity mapping to exome sequencing strategies, protein interaction networks,^8^ and genome-wide small interfering RNA screens,^9^ allowing a rapid rate of gene discovery. Despite these advancements, which made it possible for the majority of JBTS cases to have a genetic diagnosis,^10^ many cases of JBTS remain genetically unsolved, and critically, the inter-relationships between the proteins encoded by these genes and the underlying disease mechanisms remain poorly understood. Here, we used a combination of autozygosity mapping and whole-exome sequencing (WES)^11^, ^12^ in two unsolved JBTS-affected families and identified likely deleterious variants in *ARL3* (MIM: 60495). We further investigate the mechanistic impact of this mutation and show that the mutant ARL3 is irresponsive to the guanine nucleotide exchange factor (GEF) activity of ARL13B and causes associated defects in ciliary proteins in affected individuals’ fibroblasts.

**Figure 1:**
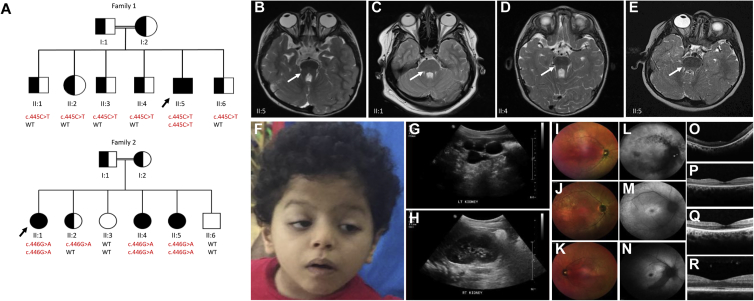
Clinical and Radiological Images of the Affected Members of the Two Families Included in This Study (A) A pedigree of the two families shows the number of affected siblings in each family and the outcome of segregation analysis (affected, shaded; carriers, half-shaded; and WT, unshaded). The proband in each family is indicated by a black arrow. Genotypes for the proband and their siblings are shown. (B–E) Brain MRI of the four affected individuals (B, II:5 in family 1; C, D, and E, II:1, II:4, and II:5 in family 2) in this study shows evidence of a molar tooth sign, cerebellar vermis hypoplasia, and elongation of the superior cerebellar peduncles (arrowed). (F) Facial photo of the proband (II:5) in family 1 shows dysmorphic features (depressed nasal bridge, upturned nares, ptosis, arched eyebrows, synophrys, telecanthus, and low-set ears). (G and H) Ultrasound scan image of the kidneys of the affected member in family 1 (II:5) shows an echogenic left multicystic dysplastic kidney (G) and an unaffected right kidney (H). (I–R) Retinal imaging, including multicolor scanning laser fundal images of the eyes, of the three affected siblings in family 2 (II:1, II:4, and II:5) shows granular alterations of the retinal pigment epithelium and subtle spicule formation, particularly around the major vascular arcades, and arteriolar attenuation (I, II:1; J, II:4; K, II:5). Autofluorescence images show stippled hypo-autofluorescence areas concentrated around the arcades (L, II:1) and hyper-autofluorescence around fovea (M, II:4; N, II:5). Horizontal optical coherence tomography scans demonstrate thinning of the outer nuclear layer and loss of ellipsoid and external limiting membrane lines with preservation of inner retinal lamination in all three siblings (O, II:1; P, II:4; Q, II:5). A horizontal optical coherence tomography scan of a healthy control individual is shown for comparison (R).

Family 1 is a Saudi Arabian family comprising first-cousin healthy parents and six children, including the 5-year-old male index individual (II:5; Figure 1). His clinical features include developmental delay, multicystic dysplastic left kidney, night blindness, and mild dysmorphic features, including ptosis (Figure 1 and Table 1). Magnetic resonance imaging (MRI) of the brain showed severe vermis hypoplasia with abnormal thick cerebellar peduncles configured in the shape of a typical molar tooth sign (Figure 1B), as well as abnormal configuration of the midbrain, thinning of the pontomesencephalic junction and midportion of the midbrain, and mild decreased brain volume with a paucity of white matter in the frontotemporal region and dilated ventricular system. This family is part of a large ciliopathy cohort (enrolled in a research protocol approved by King Faisal Specialist Hospital and Research Center research advisory council 2080006 after providing informed consent). Family 2, originating from Pakistan, is also consanguineous and comprises three affected children with a clinical syndrome in keeping with JBTS (II;1, II:4, and II:5; Table 1 and Figure 1). The eldest sibling (II:1) presented with hypotonia and psychomotor delay. Subsequently, the child developed night blindness and bilateral visual loss by 4 years of age. She also had recurrent urinary-tract infections (Table 1). Clinical investigations revealed the molar tooth sign that is typical of JBTS on brain MRI, as well as retinal dystrophy (Figure 1). The other two affected siblings (II:4 and II:5) had very similar presentations with predominating brain and retinal features (Table 1 and Figure 1). Siblings II:1 and II:4 experienced problems with thermoregulation, which implies brainstem involvement, as well as the known cerebellar defects typical of JBTS. This family was enrolled in a research protocol approved by the National Research Ethics Service (09/H0903/36) after providing informed consent.

**Table 1:** Clinical Features of JBTS in Affected Family Members

	**Family 1**	**Family 2**		
	**II:5**	**II:1**	**II:4**	**II:5**
Age (years)	5	21	12	9
Central nervous symptoms	developmental delay, ataxia	developmental delay, ataxia	developmental delay, ataxia	developmental delay, ataxia
Ocular symptoms	ptosis, rod-cone dystrophy, night blindness, bilateral visual pathway involvement	rod-cone dystrophy, night blindness, progressive visual loss	rod-cone dystrophy, night blindness, progressive visual loss	rod-cone dystrophy, night blindness, progressive visual loss, oculomotor apraxia
eGFR (mL/min/1.73 m^2^)	NA	75	>90	>90
Renal symptoms	none	recurrent UTI	none	recurrent UTI
USS renal	left multicystic dysplastic kidney, right grade I hydronephrosis	bilateral renal scarring	normal USS	unequal kidney size
Other	single palmar crease, pectus carinatum, normal ABR	thermoregulation problems, episode of transverse myelitis	thermoregulation problems, sleep apnea	none

Abbreviations are as follows: ABR, auditory brainstem response; eGFR, estimated glomerular filtration rate; NA, not available; USS, ultrasound scan; and UTI, urinary-tract infection.

Exome sequencing of the index individual in each family and variant filtering were performed as previously described.^7^ In brief, WES was performed with the TruSeq Exome Enrichment Kit from Illumina. Coding and splicing homozygous variants were considered as candidates only if they were present within the candidate locus, had a frequency < 0.1% in publicly available variant databases (1000 Genomes, NHLBI Exome Sequencing Project Exome Variant Server, and Genome Aggregation Database [gnomAD]) and a database of in-house ethnically matched exomes (Saudi Human Genome Program; totaling 2,379 exomes), and were predicted to be pathogenic *in silico*.

**Figure 2:**
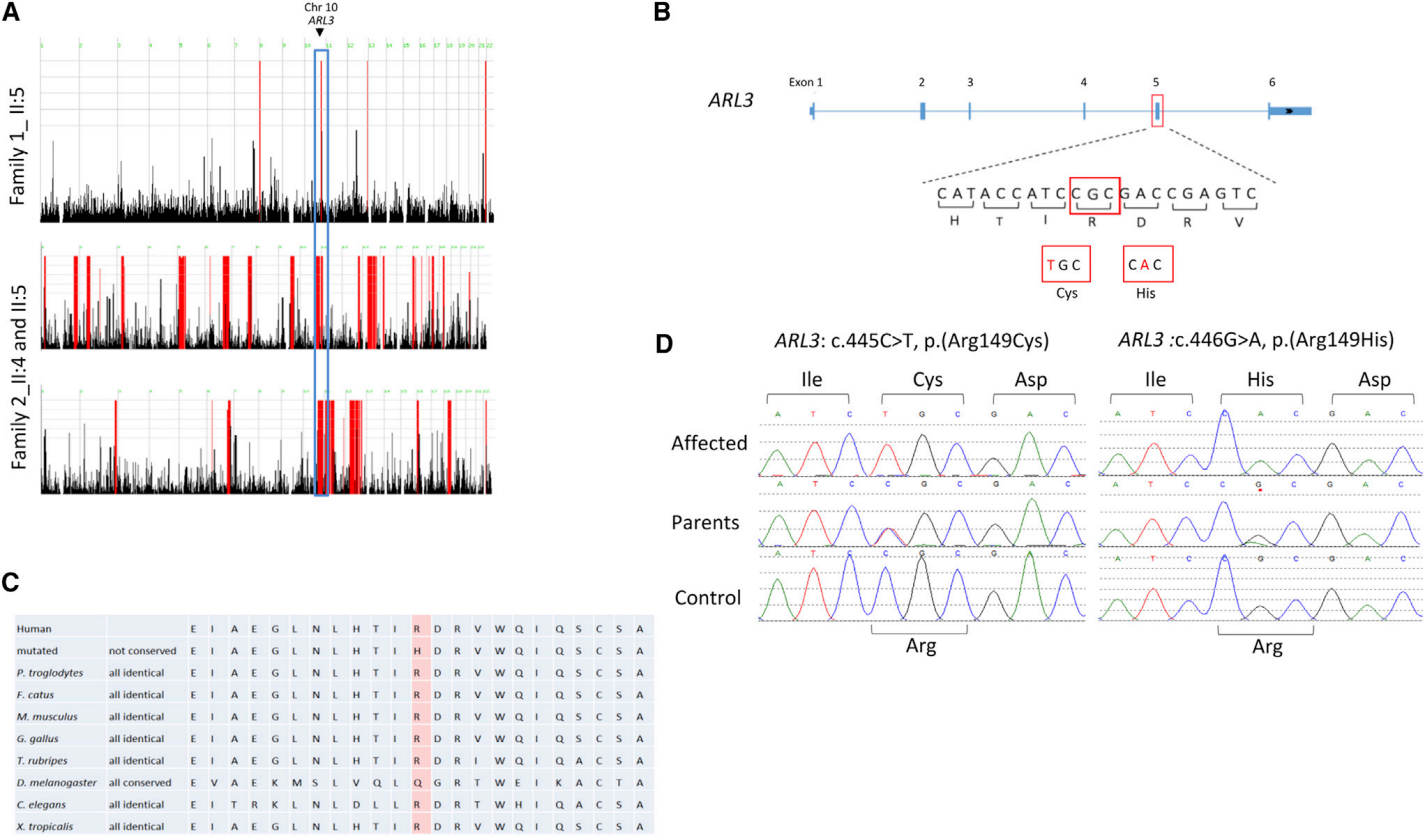
Molecular Genetic Investigations of the Two JBTS-Affected Families (A) Genome-wide homozygosity mapping shows the shared homozygous region between the affected members of the two families on chromosome 10 (blue rectangle). Regions of homozygosity are shown in red, and the position of *ARL3* is marked with a black arrow. (B) Schematic representation to *ARL3* with the homozygous missense variants located in exon 5. (C) Evolutionary conservation of residue Arg149, which is highly conserved throughout all species shown except *D. melanogaster*. (D) Sequence chromatograms of the two different *ARL3* variants described in this study.

Interestingly, both families were flagged by the corresponding research group because exome sequencing did not reveal a likely deleterious bi-allelic variant in any of the established JBTS-related genes. Through an investigator-initiated collaboration, an attempt was made to exploit the consanguineous nature of both families, which can readily reveal a potentially unifying etiology if they have an overlapping autozygome, as previously described.^7^ In brief, we performed genome-wide genotyping with the Axiom SNP Chip platform from Affymetrix and the Sure Select V4 platform from Agilent Technologies and then determined autozygomes by using HomozygosityMapper on all available family members. This revealed a single critical locus (chr10: 101,569,997–109,106,128, UCSC Genome Browser hg 19) (Figure 2A). This locus spans 57 genes, none of which is known to be linked to a ciliopathy phenotype. After re-analyzing the exome variants by only considering variants within this locus (Tables S1 and S2), we found a single previously unreported variant in *ARL3* in each index individual: c.445C>T (p.Arg149Cys) (GenBank: NM_004311.3) in family 1 and c.446G>A (p.Arg149His) (GenBank: NM_004311.3) in family 2 (Figure 2B). Both homozygous variants fully co-segregated with the JBTS phenotype in each family.

*ARL3* is a highly conserved gene, and its encoded protein, the small G-protein ARL3, localizes to the cilium and is crucial for ciliogenesis and axoneme formation, as well as cargo displacement of lipidated proteins in the cilium.^13^ *ARL3* variants have also been reported in association with retinal dystrophy.^14^ Among ARL3 effectors are the GDI-like solubilizing factors (GSFs) PDE6D, UNC119A, and UNC119B, whose interactions are guanosine triphosphate (GTP) dependent. GSFs bind to and solubilize prenylated and myristoylated proteins, which are released by ARL3-GTP acting as an allosteric release factor.^15^, ^16^ The cilia-specific protein ARL13B acts as a specific GEF for ARL3, whereas retinitis protein 2 (RP2) functions as an ARL3 GTPase-activating protein (GAP) and is localized in the pre-ciliary compartment.^17^, ^18^ This segregation of a GEF and a GAP is proposed to create an ARL3-GTP gradient inside the cilium,^19^ which ensures the destination-specific release of lipid-modified ciliary proteins, solubilized by GSFs.^20^

**Figure 3:**
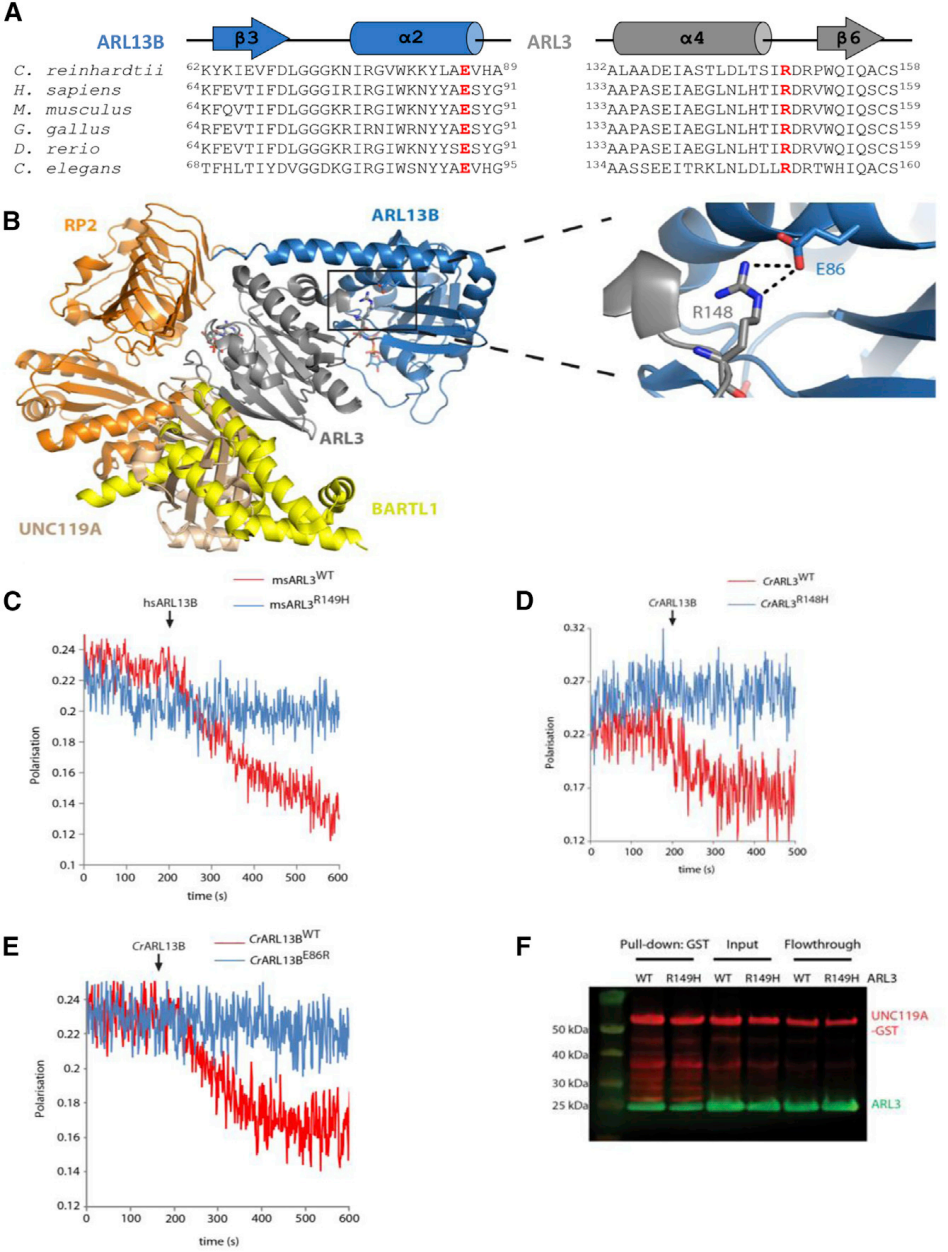
The Human ARL13B-ARL3 Complex Is Predicted to Involve an Interaction between Evolutionarily Conserved Glutamate and Arginine Residues (A) Partial amino acid sequence alignments of the ciliary GEF, ARL13B, and ARL3. Highlighted in red are the evolutionarily conserved glutamate residue located in the switch II domain of ARL13B (E86 [Glu86] in *C. reinhardtii* ARL13B [*Cr*ARL13B]) and the arginine residue in the loop region between the α4 and β6 domains of ARL3 (R148 [Arg148] in *Cr*ARL3). (B) Superimposition of the crystal structures of ARL3 (gray) in complex with its known interactors: the effectors UNC119A (salmon; PDB: 4GOJ^15^) and BARTL1 (yellow; PDB: 4ZI2^21^), the GAP RP2 (orange; PDB: 3BH6^17^), and GEF ARL13B (blue; PDB: 5DI3^19^). On the right side is a zoomed-in view of the salt bridge between Glu86 and Arg148 at the *Cr*ARL13B-*Cr*ARL3 complex interface. (C) Assay of GEF activity for murine WT ARL3 (ARL3^WT^) and p.Arg149His ARL3 (ARL3^R149H^). Fluorescence polarization was measured for 1 μM mantGDP-loaded ARL3, to which 400 μM GppNHp and 5 μM *H. sapiens* ARL13B (*Hs*ARL13B) were added. Nucleotide exchange was shown by only ARL3^WT^. (D) Assay of GEF activity with fluorescence polarization measurements of 0.5 μM mantGDP-loaded *Cr*ARL3^WT^ and *Cr*ARL3^R148H^, to which 10 μM GppNHp (GTP analog) and 5 μM *Cr*ARL13B⋅GppNHp were added at the indicated time points. Only *Cr*ARL3^WT^ showed nucleotide exchange, as indicated by the drop in fluorescence polarization. (E) Assay of GEF activity with fluorescence polarization measurements of 0.5 μM mantGDP-loaded *Cr*ARL3^WT^ and 5 μM *Cr*ARL13B⋅GppNHp^WT^ or *Cr*Arl13b^E86R^, to which 10 μM GppNHp (GTP analog) was added at the indicated time points. Only *Cr*Arl13b^WT^ showed nucleotide exchange, as indicated by the drop in fluorescence polarization. (F) 30 μg of full-length UNC119A-GST was used to pull down 60 μg of murine ARL3^WT^ and ARL3^R149H^ that were loaded with the GTP analog GppNHp. Proteins were detected on immunoblots with anti-GST (red) and anti-His (green) antibodies.

The ARL3 Arg149 residue is highly conserved throughout evolution (Figure 2C), and *in silico* prediction tools suggest that either missense change is likely to be pathogenic (Table S3). Homology models of ARL3 reveal that the two variants, which are located in a loop between the α4 and β6 domains (Figure 3A), are predicted to disrupt the interaction of ARL13B with ARL3 because it requires this precise residue (Arg149) for its interaction (Figure 3B). Superimposing all known structures of ARL3 in complex with its effectors, GAP and GEFS, the ARL3 Arg149 residue is exclusively present in the interface between ARL3 and ARL13B and is involved in an ionic interaction with the conserved ARL13B Glu88 residue (Figure 3B). To functionally investigate the effect of the mutation on the interaction with ARL13B, we performed a GEF fluorescence-based polarization experiment.^19^ Wild-type (WT) and mutant p.Arg149His versions of murine ARL3 (98.35% sequence identity to human ARL3) were bound to fluorescently labeled GDP, and an excess of unlabeled GTP was added in the presence or absence of human ARL13B. We then followed the capability for nucleotide exchange of both versions of the protein by recording the fluorescence polarization over time. Upon addition of the ARL13B GEF, WT ARL3 showed a clear acceleration of nucleotide exchange. Under similar conditions, mutant p.Arg149His ARL3 failed to show acceleration of nucleotide exchange in the presence of ARL13B (Figure 3C). The integrity of the mutant protein was confirmed by pull-down, whereby both WT and p.Arg149His ARL3 proteins were pulled down equally by UNC119A (Figure 3F and Figure S1). Furthermore, we confirmed our results by using the highly conserved *C. reinhardtii* ARL3 (WT and mutant p.Arg148His) and ARL13B (Figure 3D). To further investigate the importance of the ARL3-ARL13B interaction, we carried out the reverse charge variant p.Glu86Arg in ARL13B by using *C. reinhardtii* proteins. As expected, p.Glu86Arg ARL13B was not able to accelerate the nucleotide exchange of WT ARL3 (Figure 3E). From these experiments, we conclude that p.Arg149His ARL3 disrupts the interaction with ARL13B and is defective in ARL13B-assisted nucleotide exchange.

To determine ciliary morphology, we obtained fibroblasts from all three affected individuals in family 2 (II:1, II:4, and II:5) plus control individuals (both parents [I:1 and I:2] and an unaffected sibling [II:3]). Primary cilia identified by ARL13B antibodies were of normal length in affected individuals (mean length = 5.9, 7.8, and 6.8 μm in II:1, II:4, and II:5, respectively) and control individuals (mean length = 5.7 and 6.0 μm in the parents and 6.1 μm in the unaffected sibling), and there were no significant differences between the two groups (Figure S2). There was also no difference in the percentage of ciliation rates between affected and control fibroblasts (Figure S2). Scanning electron microscopy confirmed these findings of no significant changes in cilia length or structural appearance (Figure S3).

**Figure 4:**
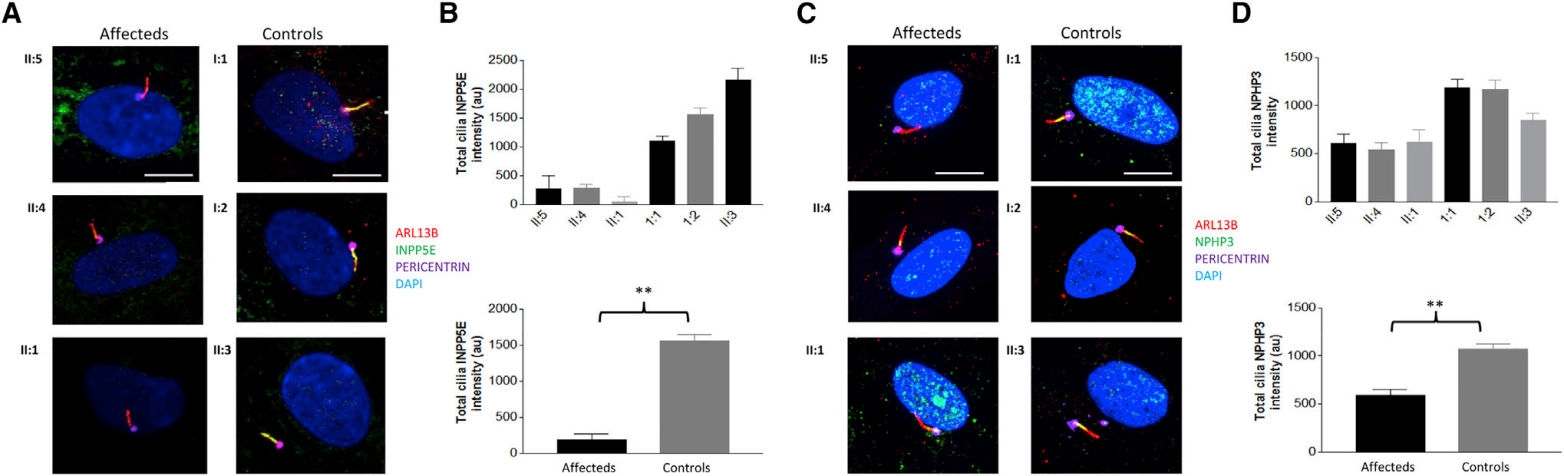
Characterization of Ciliary Phenotype in *ARL3*-Mutant Fibroblasts from Family 2 (A and C) Affected and control fibroblasts were observed under high-power immunofluorescence for determining ciliary expression of (A) INPP5E and (C) NPHP3. Cilia were localized with anti-ARL13B (red) and anti-PERICENTRIN (magenta) for the identification of the ciliary membrane and the base of cilia, respectively. Scale bars, 10 μm. (B) Quantification of ciliary localization of INPP5E (^∗∗^p < 0.0001, unpaired t test, n > 150 cilia for each group). Total cilia INPP5E in control fibroblast (II:3) cilia is higher than in heterozygous fibroblast (I:1 and II:2) cilia. (D) Quantification of ciliary localization of NPHP3 (^∗∗^p < 0.0001, unpaired t test, n > 150 cilia for each group).

ARL3 functions as an allosteric release factor of all GSFs members: PDE6D, UNC119A, and UNC119B. Whereas PDE6D is involved in the trafficking of prenylated proteins, UNC119A and UNC119B traffic myristoylated proteins.^19^ Given that ARL3 exerts its releasing function only when bound to GTP, we expected the ciliary localization of the GSF cargo to be impaired. The INPP5E, GRK1, and PDE6 catalytic subunits are among the prenylated GSF ciliary cargo,^20^ whereas the myristoylated ciliary cargo includes NPHP3, GNAT1, and Cystin1.^22^ To test our hypothesis, we examined cilia for protein content of both the prenylated INPP5E and myristoylated NPHP3. *ARL3*-mutant cilia demonstrated a significant loss of both INPP5E and NPHP3 content (Figure 4 and Figures S4–S6), indicating that WT ARL3 is required for normal release of these cargos into the ciliary axoneme. To confirm these phenotypes as specific to the loss of ARL3 function, we sought to determine the ciliary content of GLI3 in WT and ARL3-mutant cilia. GLI3 translocation is independent of GSF transport and relies upon intraflagellar transport proteins and Sonic Hedgehog signal transduction.^23^ Consistent with morphologically normal cilia in *ARL3*-mutant fibroblasts, no defect in ciliary GLI3 was observed after stimulation with SAG, a Hedgehog pathway agonist. The amounts of total ciliary GLI3 and ciliary tip GLI3 were unchanged between affected and control individuals (Figure S7), confirming that the ciliary Hedgehog signaling pathway is not disturbed by this particular *ARL3* mutation. Together, these data substantiate a role for ARL3 in the release of both prenylated and myristoylated ciliary cargo, which is disrupted by the p.Arg149His ARL3 variant.

We present *ARL3* as a ciliopathy- and JBTS-associated gene. *Ld*ARL-3A, a *Leishmania* homolog of ARL3, is an essential component of flagellum formation.^24^ *Arl3* knockdown has previously been investigated in a gene-trap murine model, where *Arl3* was disrupted after the first exon.^25^ These *Arl3*^−/−^ mice, which represent a null allele, developed a severe ciliopathy phenotype with pronounced cystic kidney disease, pancreatic hypoplasia, ductal plate malformation within the liver, and retinal dystrophy with impaired photoreceptor development.^25^ The mice died within 3 weeks of age, indicating a severe phenotype, which is much more detrimental than that of our human subjects, who carry a missense mutation. We speculate that nonsense mutations in *ARL3* in humans could cause more pronounced ciliopathy phenotypes, such as the perinatally lethal ciliopathy Meckel syndrome,^26^ and could go some way to explaining why such a fundamental gene has previously not been identified in ciliopathy syndromes. It is noteworthy that the ExAC Browser and gnomAD do not have any homozygous pathogenic variants reported within *ARL3* and that the gene is relatively intolerant to variation (positive *Z* score of 0.44). We did not identify any additional *ARL3* pathogenic variants in our WES databases, which are relatively enriched with autozygosity, or in a cohort of 35 unsolved JBTS-affected individuals.

In humans, Strom et al. previously reported the heterozygous missense variant c.269A>G (p.Tyr90Cys) in *ARL3* in a European-descent pedigree with non-syndromic retinitis pigmentosa.^27^ The variant, which was rare, appeared *de novo* and was predicted to be pathogenic, was confirmed as heterozygous in three affected individuals, and was transmitted in an autosomal-dominant fashion. A second allele was not identified, and mechanistic evaluation was not carried out. On the other hand, here we have identified bi-allelic *ARL3* changes that fully segregate with a classical JBTS phenotype, including retinal changes. Thus, although the connection between the *de novo ARL3* variant and retinitis pigmentosa remains unexplained, it seems that bi-allelic *ARL3* deleterious variants are sufficient to cause JBTS. The involvement of ciliopathy-associated genes in non-syndromic retinitis pigmentosa has been well described, so it would be of interest for the affected individual reported by Strom et al. to be investigated for the possibility of a second deleterious allele in *trans* in *ARL3*. It is also possible that, as reported here, bi-allelic mutations in *ARL3* give rise to an extended phenotype compared with its reported dominant phenotype. A growing number of genes are known to cause distinct phenotypes according to whether dominant or recessive variants are inherited. For retinitis pigmentosa, mutations (typically nonsense) in *RP1* were initially described in an autosomal-dominant pattern,^28^ followed by autosomal-recessive (homozygous missense) variants.^29^ For Gillespie syndrome, a form of non-progressive cerebellar ataxia, both bi-allelic and mono-allelic mutations in *ITPR1* (MIM: 147265) have been reported,^30^ and the single heterozygous mutations were thought to exert a dominant-negative effect. In addition, variants in genes known only to be related to autosomal-dominant disease have been found in association with recessive mutations, both where the phenotypes are similar but more severe (*ACTG2*-related visceral myopathy [MIM: 102545]) and where distinctly different phenotypes have been observed (*FBN2*-related myopathy [MIM: 612570] and *CSF1R*-related brain malformation [MIM: 164770]).^31^

Interestingly, pathogenic variants in the ARL3 interaction partners ARL13B and PDE6D also cause JBTS. *ARL13B* (MIM: 608922) mutations were reported in individuals with a classical neurodevelopmental JBTS phenotype (JBTS8) without prominent renal phenotypes.^32^ It is particularly noteworthy that some affected individuals had a small occipital encephalocele, indicating that more severe brain phenotypes could be likely. *PDE6D* (MIM: 602676) mutations have been reported in three siblings with JBTS (JBTS22) and associated retinal and post-axial polydactyly phenotypes, as well as kidney hypoplasia.^33^ Furthermore, the disrupted ciliary cargo proteins (INPP5E and NPHP3) we identified are also responsible for JBTS phenotypes when their encoding genes are mutated. *INPP5E* (MIM: 613037) mutations cause JBTS1 and were identified in a cohort of JBTS-affected individuals with mainly neurological features and some retinopathy but without kidney disease or polydactyly,^34^ suggesting a lack of Hedgehog signaling defects.^35^, ^36^ In contrast, *NPHP3* (MIM: 608002) mutations were initially identified as causing an adolescent form of nephronophthisis, a progressive form of renal failure.^37^ Since this initial report, *NPHP3* mutations have been associated with a wider spectrum of disease, including infantile nephronophthisis (resulting in end-stage renal failure before 5 years of age^38^) and Meckel syndrome.^39^ The full spectrum of disease phenotypes secondary to *ARL3* mutation therefore remains to be determined, but *ARL3* is widely expressed and fundamental to the ciliary localization of a wide range of proteins. Therefore, one could predict that any severity of JBTS disease with retinal and renal involvement is possible. It will be important to study the tissue-specific roles of *ARL3* and the implications of disrupting expression in these tissues.

**Figure 5:**
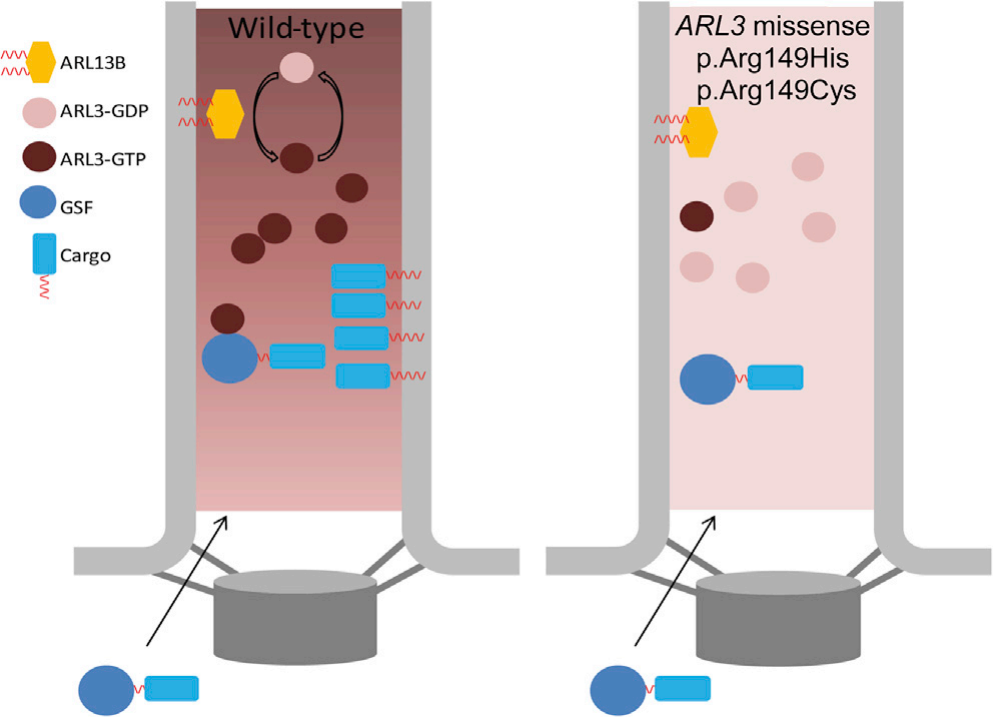
Model of GSF-Cargo Release in Cilia with WT ARL3 versus p.Arg149 ARL3 Missense Variants ARL13B assists ARL3 in cilia to exchange its bound GDP to GTP. The specific localization of ARL13B in the cilia creates a high concentration of ARL3-GTP. ARL3-GTP in turn can release the cargo bound to its cognate GSF, resulting in ciliary localization. Missense variants of ARL3 (including p.Arg149His and p.Arg149Cys) are not able to interact with ARL13B, and the ARL3GTP concentration is therefore low in the cilia, resulting in inefficient release of GSF cargo in the cilia.

The primary cilium exerts its function by concentrating certain proteins and lipids, thereby maintaining a distinct composition and function. ARL13B is specifically localized in the cilium, creating a high ciliary concentration of ARL3-GTP, which in turn produces a hotspot for releasing GSF-bound cargo. Our study underscores the physiological importance of this mechanism because the human mutation we characterize, c.446G>A (p.Arg149His), disrupts the interaction between ARL13B and ARL3 and results in loss of GSF cargo concentration in the cilia (Figure 5). As we have described, ciliopathies such as JBTS show overlapping phenotypes, and one gene can be involved in a broad range of ciliopathy phenotypes. A cause of this overlap is most likely the fact that proteins do not work in isolation but in networks. Indeed, it has been shown that ciliopathy-associated proteins form different modules that cross talk and interact together.^8^, ^40^ Through the identification of *ARL3* variants as a cause of JBTS, we show that ARL3 provides a hub within the network of ciliopathy-associated genes, whereby perturbation of ARL3 results in the mislocalization of multiple ciliary proteins, including INPP5E and NPHP3. Our mechanistic model might provide good starting points for therapeutic intervention where small molecules can be used to release GSF-bound cargo and compensate for the loss of ARL3 release activity. Directing such therapies to the kidney in individuals with JBTS-associated renal dysfunction and to the retina in cases of progressive visual loss secondary to JBTS would be desirable. Using small molecules to disrupt GSF-cargo interaction has been reported,^41^, ^42^ and it will be important to test those small molecules with regard to cilia function and their application in ciliopathies. Nevertheless, a challenge will be to target these small molecules specifically to the cilia, where the function of ARL3 is concentrated, to assure the correct targeting of ciliary GSF cargo.

In conclusion, we have identified *ARL3* missense variants as a likely cause of JBTS. On the basis of limited observations, the phenotype related to variants in this gene seem to be a cerebello-retinal presentation similar to that caused, for instance, by pathogenic variants in *AHI1* (MIM: 608894).^43^, ^44^ In fact, none of the affected individuals presented with any striking additional features, and renal involvement was inconstant. Because effective treatments for JBTS are lacking at present, genotype-phenotype correlations could prove useful in giving prognostic indications to families. We have shown that substitution of arginine at position 149 disrupts the known interaction between ARL3 and ARL13B and thus prevents the correct release of intra-ciliary cargos, including INPP5E and NPHP3. Furthermore, we propose that therapeutic manipulation of ciliary cargo release could provide an innovative treatment mechanism for human ciliopathies such as JBTS.

## Declaration of Interests

The authors declare no competing interests.

## Accession Numbers

Gene variants reported in this manuscript (for subjects 00179519, 00179520, 00179521, and 00179522) have been submitted to LOVD at https://databases.lovd.nl/shared/genes/ARL3.

## Web Resources

Combined Annotation Dependent Depletion (CADD), http://cadd.gs.washington.edu/
ExAC Browser, http://exac.broadinstitute.org/
GenBank, https://www.ncbi.nlm.nih.gov/genbank/
GeneReviews, Parisi, M., and Glass, I. (1993). Joubert Syndrome, https://www.ncbi.nlm.nih.gov/books/NBK1325/
Genome Aggregation Database (gnomAD), http://gnomad.broadinstitute.org/
HomozygosityMapper, http://www.homozygositymapper.org/
OMIM, https://www.omim.org/
Leiden Open Variation Database (LOVD), https://databases.lovd.nl/shared/genes/ARL3
MutationTaster, http://www.mutationtaster.org/
RSCB Protein Data Bank, https://www.rcsb.org/pdb/home/home.do
UCSC Genome Browser, https://genome.ucsc.edu/

## Supplemental Data

Document S1. Supplemental Material and Methods, Figures S1–S7, and Tables S1–S3

Document S2. Article plus Supplemental Data
