# Supplementary material for: ARL3 Mutations Cause Joubert Syndrome by Disrupting Ciliary Protein Composition
Source: Am J Hum Genet. 2018 Sep 27;103(4):612–20. doi: 10.1016/j.ajhg.2018.08.015 (PMC6174286; doi:10.1016/j.ajhg.2018.08.015)
Supplement: Document S1. Supplemental Material and Methods, Figures S1–S7, and Tables S1–S3 [file mmc1.pdf]

The American Journal of Human Genetics, Volume *103*

Supplemental Data

ARL3 Mutations Cause Joubert Syndrome by Disrupting Ciliary Protein Composition

Sumaya Alkanderi, Elisa Molinari, Ranad Shaheen, Yasmin Elmaghloob, Louise A. Stephen, Veronica Sammut, Simon A. Ramsbottom, Shalabh Srivastava, George Cairns, Noel Edwards, Sarah J. Rice, Nour Ewida, Amal Alhashem, Kathryn White, Colin G. Miles, David H. Steel, Fowzan S. Alkuraya, Shehab Ismail, and John A. Sayer

Supplemental Figures

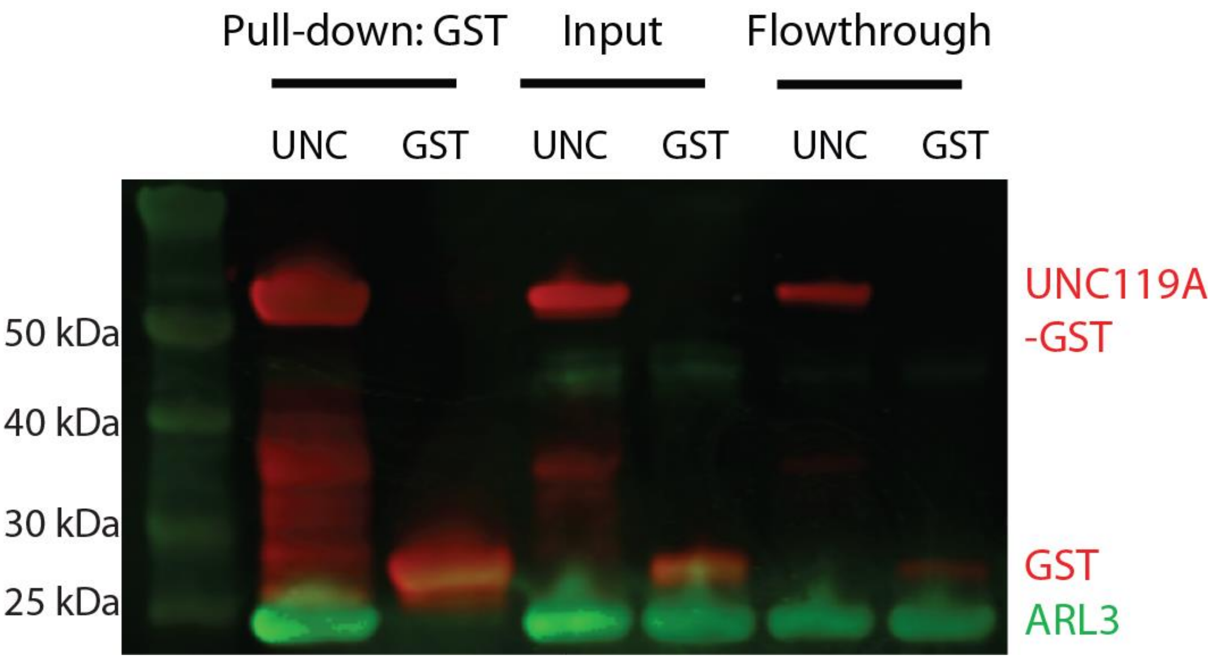

Figure S1. ARL3 specifically interacts with UNC119A

30 µg of full-length UNC119A-GST (labelled UNC) or GST alone (labelled GST) were used to pull down 60 µg of murine ARL3^{WT} that was loaded with the GTP analogue GppNHp. Proteins were detected on immunoblots using anti-GST (red) and anti-His (green) antibodies.

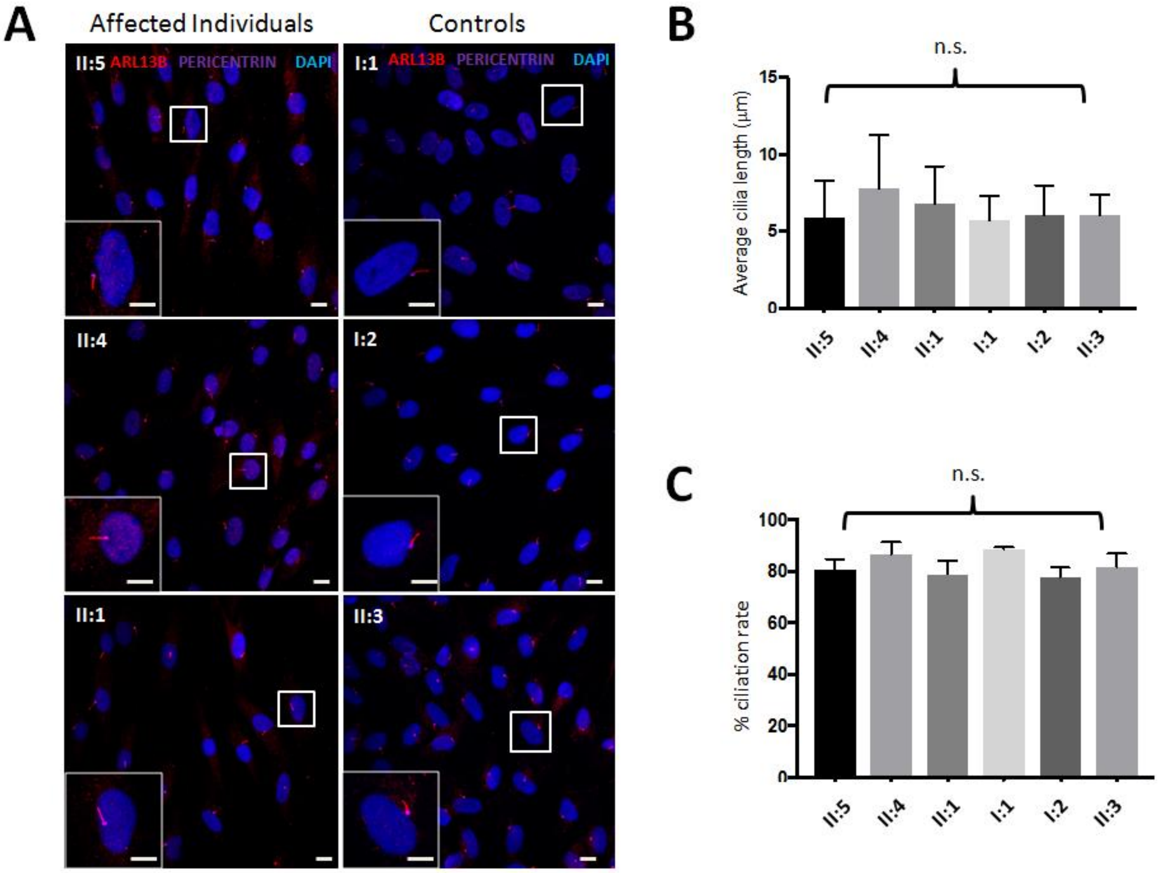

Figure S2. Analysis of cilia length using immunofluorescence in affected individuals and unaffected relative fibroblasts

(A) Fibroblasts from affected individuals in family 2 (II:5, II:4, II:1) and controls (I:1, I;2, II:3) were imaged using immunofluorescence microscopy following staining with anti-ARL13B (red) to identify ciliary membrane, anti-PERICENTRIN (magenta) to identify the ciliary base and DAPI (blue) to identify the cell nucleus. Scale bar = 10 μm.

(B) Quantification of cilia length from affected and control fibroblasts (n.s. not significant, ANOVA).

(C) Percentage ciliation rate in affected and control fibroblasts (n.s. not significant, ANOVA).

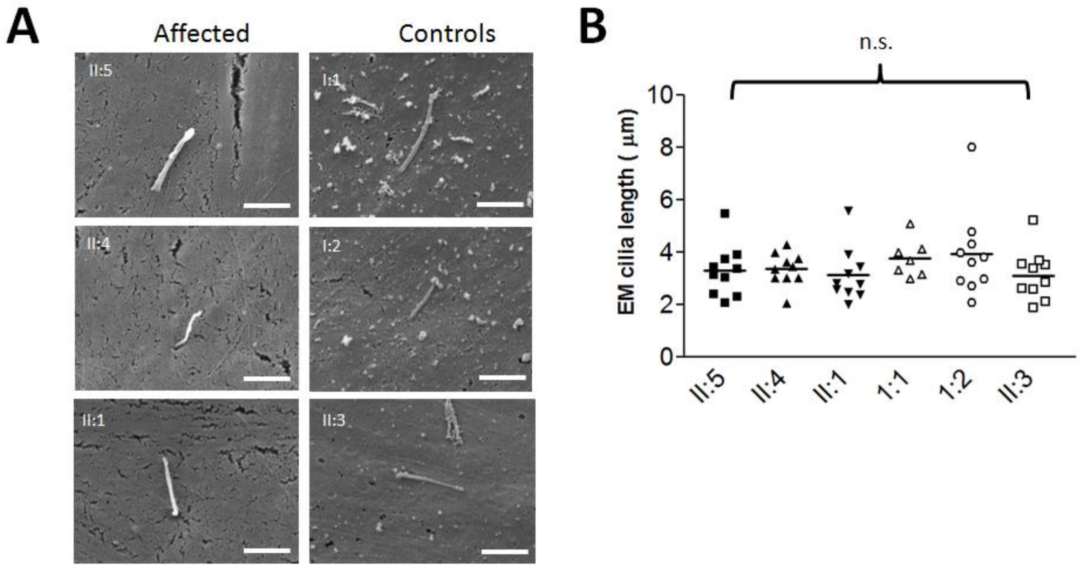

Figure S3. Scanning electron microscopy and quantification of cilia length in *ARL3* mutant and control fibroblasts

(A) Fibroblasts from affected individuals in family 2 (II:5, II:4, II:1) and controls (I:1, I;2, II:3) seen under scanning EM reveal normal appearances of primary cilia (Scale bar 2 µm).

(B) Dot plot to show quantification of EM cilia length, bars represent mean (n.s. not significant, ANOVA).

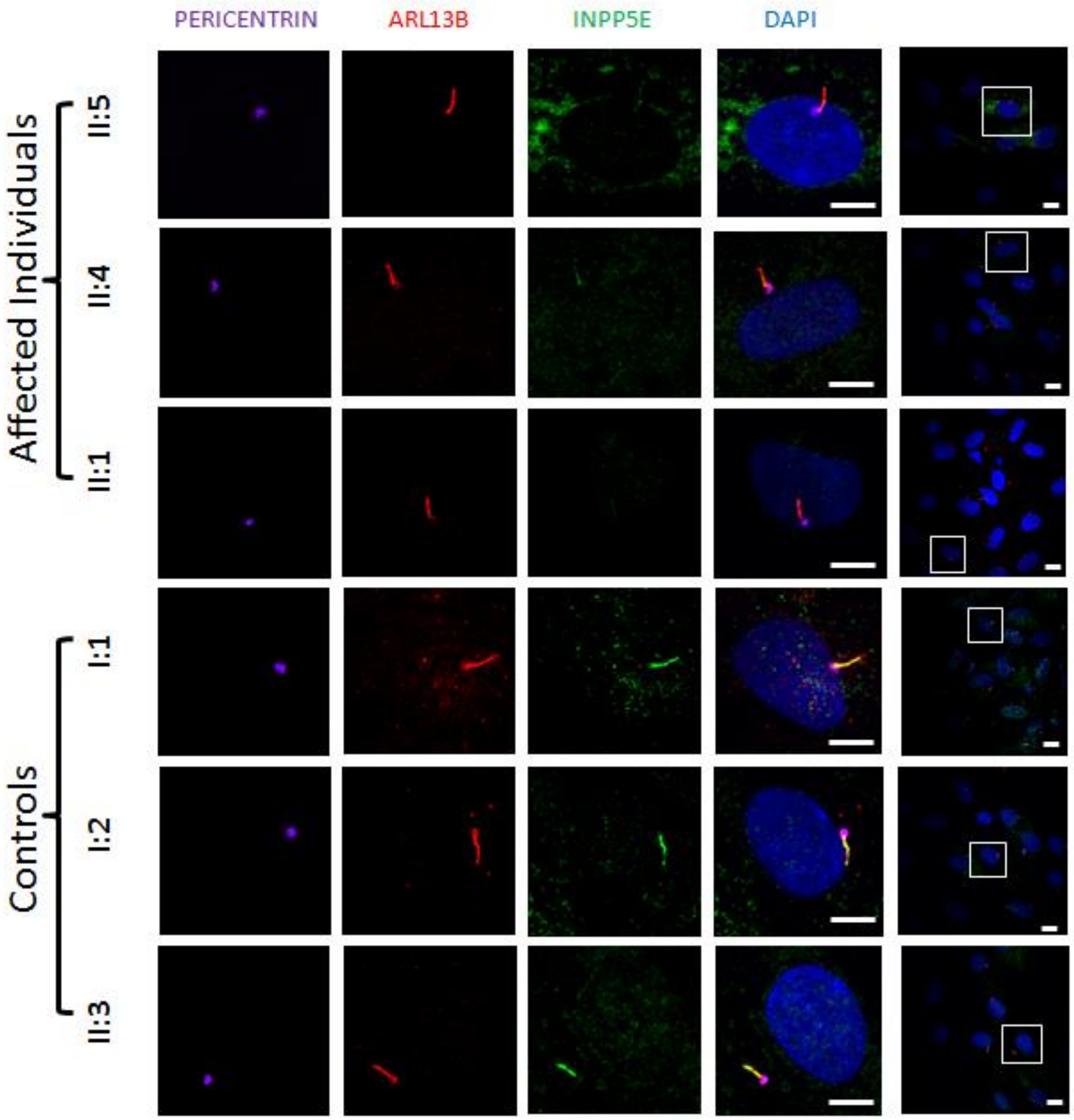

Figure S4. Analysis of INPP5E content in fibroblast cilia

Fibroblasts from affected individuals in family 2 (II:5, II:4, II:1) and controls (I:1, I:2, II:3) were fixed and stained with antibodies directed towards PERICENTRIN (magenta), ARL13B (red), INPP5E (green) and DAPI (blue) as a nuclear marker. Representative individual images are shown for PERICENTRIN, ARL13B and INPP5E as well as overlay images at high and low power. Scale bar = 10 µm.

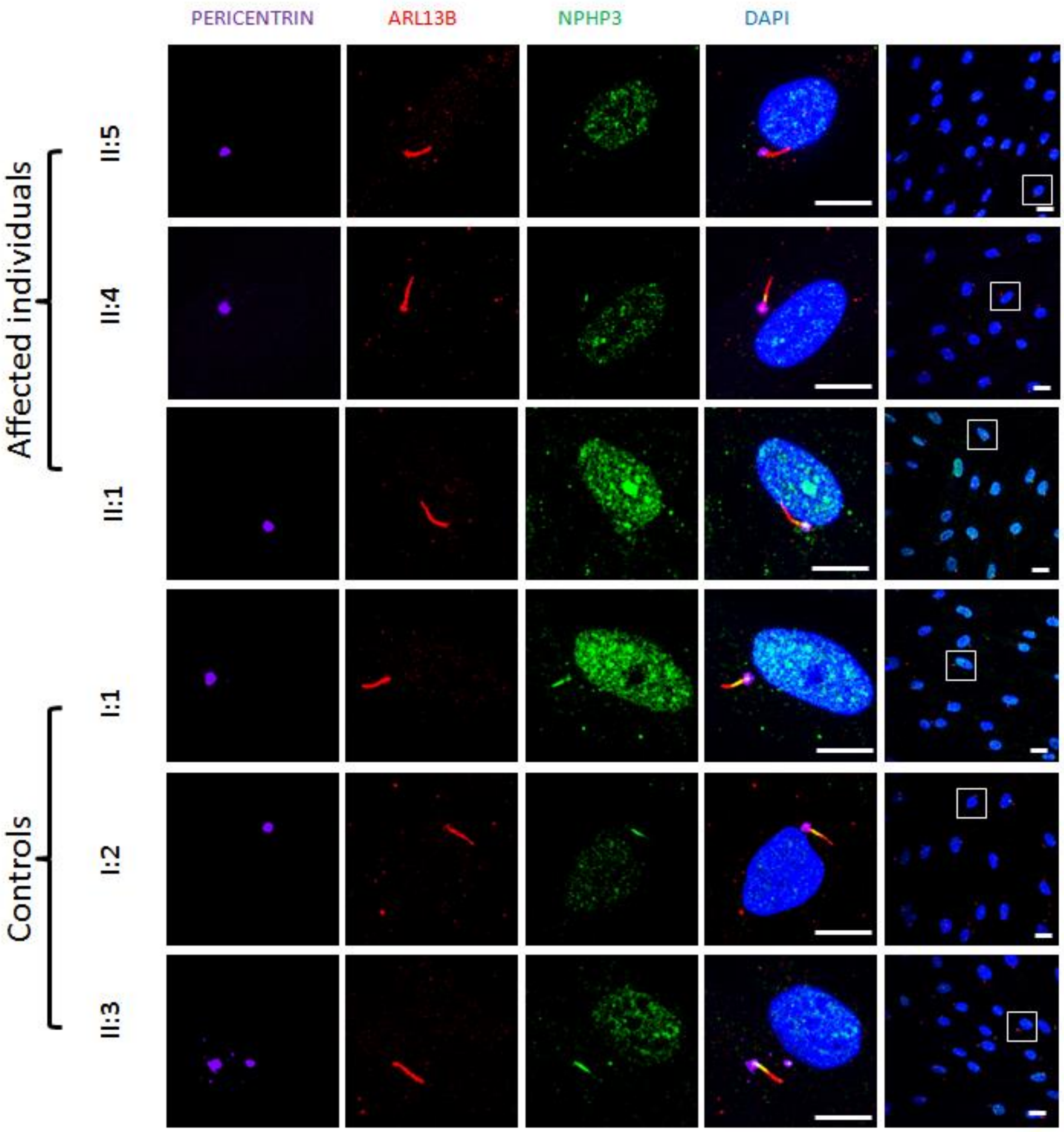

Figure S5. Analysis of NPHP3 content in fibroblast cilia

Fibroblasts from affected individuals in family 2 (II:5, II:4, II:1) and controls (I:1, I:2, II:3) were fixed and stained with antibodies directed towards PERICENTRIN (magenta), ARL13B (red), NPHP3 (green) and DAPI (blue) as a nuclear marker. Representative individual images are shown for PERICENTRIN, ARL13B and NPHP3 as well as overlay images at high and low power. Scale bar = 10 µm. Note, intracellular NPHP3 staining (green) is variable, does not correspond to genotype and may be non-specific.

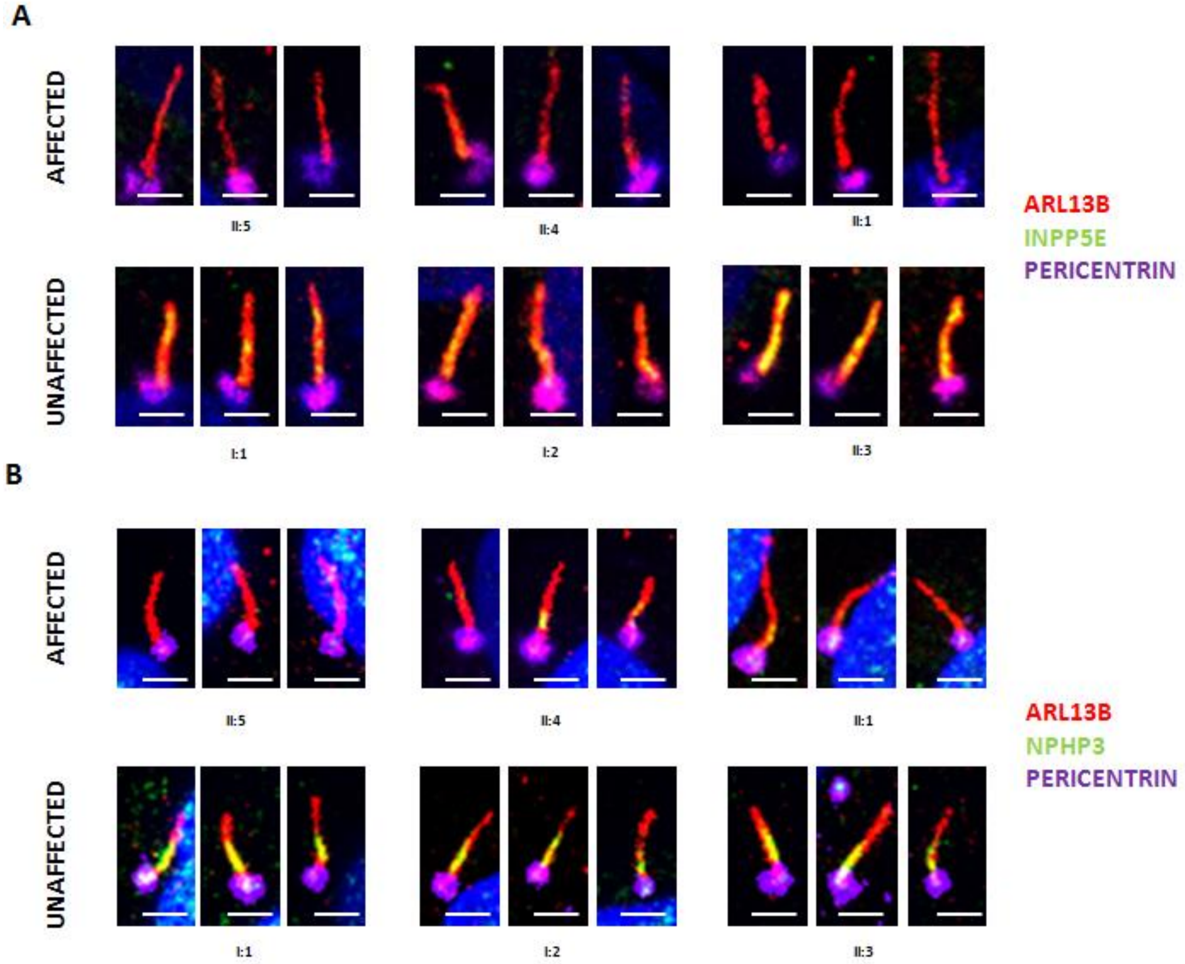

Figure S6. INPP5E and NPHP3 content in fibroblast cilia

Zoomed overlay images of fibroblast primary cilia from affected individuals in family 2 (II:5, II:4, II:1) and controls (I:1, I:2, II:3) were fixed and stained with antibodies directed towards PERICENTRIN (magenta), ARL13B (red), and (A) INPP5E (green) and (B) NPHP3 (green) and DAPI (blue) as a nuclear marker.

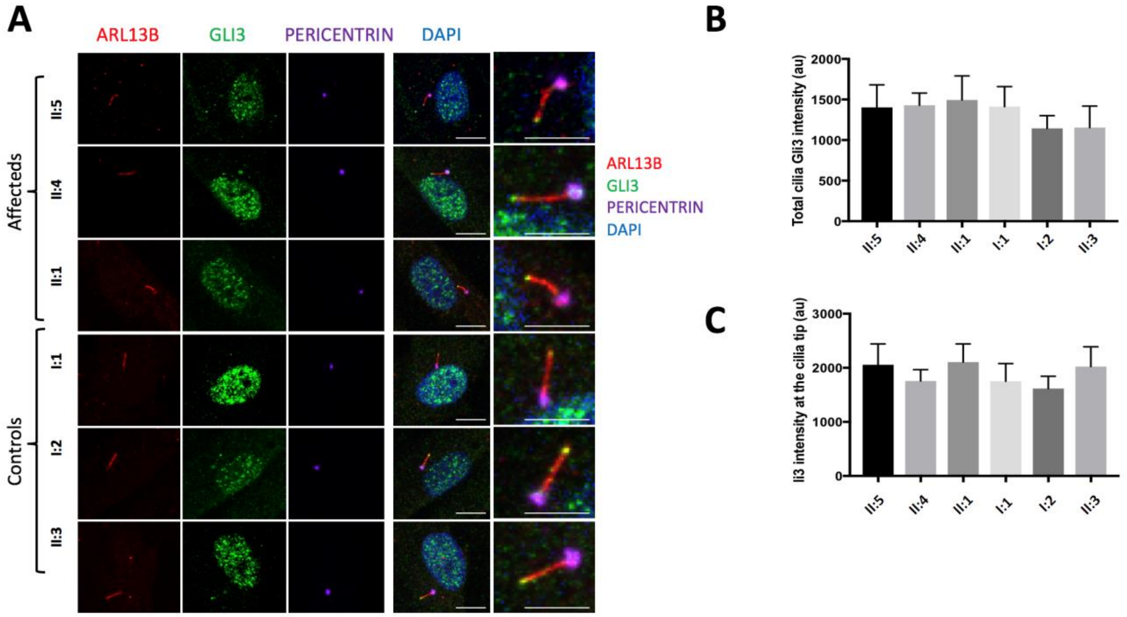

Figure S7. Lack of disruption of GLI3 localisation in cilia axoneme and tip in ARL3 mutant fibroblasts

(A) Fibroblasts from affected individuals in family 2 (II:5, II:4, II:1) and controls (I:1, I:2, II:3) were treated with 100 nM SAG then fixed and stained with antibodies directed towards ARL13B (red), GLI3 (green) PERICENTRIN (magenta) and DAPI (blue) as a nuclear marker. Representative single channel images are shown for ARL13B, GLI3 and PERICENTRIN as well as overlay images at low power (scale bar = 10 µm) and zoomed (scale bar = 5 µm) .

(B) Quantification of ciliary localisation of GLI3 is shown (n >35 for each group, n.s. not significant, ANOVA).

(C) Quantification of ciliary tip localisation of GLI3 is shown (n >35 for each group, n.s. not significant, ANOVA).

Supplementary tables

	Family 1	Family 2	
Affected member	II:5	II:4	II:5
Number of variants	52809	145724	144048
Homozygous coding / splicing < 0.1% in 1000 Genome database, gnomAD, ExAC and in-house ethnically matched exomes	16	42	31
Within the homozygous regions	11	8	15
Predicted to be pathogenic	2	5	3
Biological Context (Known JBTS genes)	0	0	0
Within the shared ROH (3 affected)	1	1	1

Table S1. Filtering criteria to the WES files of the three affected members from the two families included in this study.

Family 1 II:5	Reason for inclusion /exclusion
**Chr10.** ***ARL3*****:** NM_004311.3: c.445C>T, p.(Arg149Cys)	Include: candidate ciliopathy gene
**Chr8.** ***MTMR7*****:** NM_004686.4:c.547C>T, p.(Arg183Trp)	Exclude: It encodes a myotubularin related protein. Mutation in *MTMR1*, which encode a similar member of the myotubularin related family of proteins cause Charcot-Marie-Tooth neuropathies.
Family 2 II:4	
**Chr10.** ***ARL3*** NM_004311.3: c.446G>A, p.(Arg149His)	Include: Candidate ciliopathy gene
**Chr11.** ***PKP3*** NM_007183.3: c.649C.T, p.(Arg217Cys)	Exclude: encodes a desmosomal plaque protein. Variant not found in sibling or Family 1
**Chr11.** ***OR52N4*** NM_001005175.3: c.656C>T, p. (Ser219Phe)	Exclude: Olfactory Receptor family member. Variant not found in sibling or Family 1
**Chr13.** ***FAM124A*** NM_145019.3: c.1717G>T, p.(Gly573Trp)	Exclude: Family With Sequence Similarity 124 Member A. Variant not found in sibling or Family 1
**Chr17.** ***SLC5A10*** NM.001042450.2, c.1412G>A, p.(Gly435Glu)	Exclude: Sodium/glucose transporter, expression limited to kidney. Variant not found in sibling or Family 1
Family 2 II:5	
**Chr7.** ***CTAGE9*** NM_001008747.2, c.306G>C, p.(Glu102Asp)	Exclude: cutaneous T-cell lymphoma-associated antigen 9. Variant not found in sibling or Family 1
**Chr10.** ***ARL3*** NM_004311.3: c.446G>A, p.(Arg149His)	Include: Candidate ciliopathy gene
**Chr12.** ***DPY19L2*** NM_173812.4, c.1660A>G, p.(Met554Cys)	Exclude: known phenotype of spermatogenic failure (MIM 613893). Variant not found in sibling or Family 1

Table S2. Predicted Pathogenic Homozygous Variants Found

Nucleotide change (Ref sequence NM_004311.3)	Amino acid change	Mutation Taster	PolyPhen 2	SIFT	CADD Score	gnomAD / ExAC allele frequency
c.445C>T	p.(Arg149 Cys)	Disease Causing	Probably damaging (0.912)	Deleterious (0)	35	0.0000325 / 0.00003298
c.446G>A	p.(Arg149 His)	Disease Causing	Possibly damaging (0.758)	Deleterious (0.02)	34	0.000004063/ 0.00000824

Table S3. ***In silico*** **analysis of the** ***ARL3*** **mutations**

CADD Score, Combined Annotation Dependent Depletion, score >20 indicates the 1% most deleterious amino acid substitution

Supplemental materials and methods

Ethics and informed consent

All affected individuals and their relatives consented to this study. For Saudi Arabian individuals, ethical approval and informed consent was obtained under an IRB-approved research protocol (KFSRHC RAC #2080006)). For UK individuals, ethical approval was obtained from the UK National Research Ethics Service (NRES) Committee Northern and Yorkshire (09/H0903/36) and NRES Committee North East – Newcastle and North Tyneside 1 (08/H0906/28+5). Following informed and written consent, blood samples were obtained from affected individuals and their relatives and healthy gender and age-matched controls. Skin fibroblasts were obtained from affected individuals and their relatives following informed written consent and stored in Newcastle MRC Centre Biobank for Neuromuscular and Rare Diseases.

Whole exome sequencing and analysis

Genomic DNA (2µg) from two affected siblings and their parents was subjected to whole exome paired-end sequencing analysis. Ingenuity Variant Analysis web-based application and HomozygosityMapper (http://www.homozygositymapper.org/) were used for data analysis. The variants of interest were further tested for likely pathogenicity using *in silico* prediction tools. The variants were confirmed using Sanger sequencing in all available family members to confirm segregation with disease status. Gene variants have been submitted to www.LOVD.nl/ARL3 (patient IDs 00179519, 00179520, 00179521, 00179522).

Homology modelling

Human ARL3 (UniProt accession code P36405) and ARL13B (UniProt accession code Q3SXY8) were modelled on the crystal structure of the Arl3-Arl13B complex from *Chlamydomonas reinhardtii* (PDB accession code 5DI3) using HHPred [1] and Modeller [2] software. Structures were visualised and figures prepared using PyMOL (http://www.pymol.org/).

Fibroblast culture, immunofluorescence imaging and quantification

Fibroblasts were isolated from skin biopsies, cultured in DMEM, 10% foetal bovine serum (FBS) and 1% Pen/Strep and incubated in 37 °C. For cilia imaging, cells were seeded on coverslips and grown to 100% confluency then starved for 48 h (in FBS free media). For GLI3 ciliary trafficking studies cells were treated with 100 nM Smoothened Agonist (SAG) after 24 h of starvation then incubated for another 2 h in FBS

free media. Cells were fixed with 100% ice-cold methanol for 15 min, then washed three times with phosphate-buffered saline (PBS). Fixed cells were incubated in PBS with 10% bovine serum albumin (BSA), as a blocking agent. For indirect immunostaining of primary cilia, primary antibodies were diluted in blocking solution. Cells were incubated in INPP5E 1:100 (Proteintech 177197-1-AP), GLI3 1:200 (Abcam ab69838) and PERICENTRIN 1:1000 (Abcam ab4448) at room temperature for 1 h or in NPHP3 1:100 (Proteintech 22026-1-AP) at 4 °C overnight. For secondary staining, cells were incubated at room temperature for 1 h with Alexa Fluor conjugated secondary antibodies diluted in blocking solution. For direct immunostaining of primary cilia, Zenon Alexa Fluor 555 Rabbit IgG labelling kit (Thermo Fisher Z25305) was used, with primary antibody ARL13B 1:400 (Proteintech 17711-1AP) diluted 1:3 in PBS with 5ul of labelling mix. Coverslips were mounted on glass slides using mounting medium with DAPI (Vectashield). Images were obtained with confocal microscopy (NIKON A1), using z-stacks and identical laser intensity as well as camera settings and imported as TIFF files. Image analysis was performed using Fiji (ImageJ) software.

For quantification of percentage of cilia, five 60X fields were analysed (nuclei vs cilia identified) then a mean score was calculated. The segmented line tool within Fiji (ImageJ) was used to measure cilia length from base of axoneme (labelled with PERICENTRIN) to the distal cilia tip. For antibody signal intensity quantification experiments, 4-5 60X fields were analysed. The freehand tool was used to mark cilia borders using ARL13B signal and a region of interest (ROI) was constructed to measure the mean signal intensity of INPP5E, NPHP3 or GLI3. Then ROI was duplicated and dragged to nearby area to correct for local background fluorescence intensity by simple subtraction. For cilia tip GLI3 signal intensity, freehand tool was used to mark cilia tip borders, mean signal intensity of labelled area (0.5-1.0 micron) was measured then corrected for local background intensity.

Scanning electron microscopy imaging

For scanning electron microscopy, cells were fixed overnight in 2% glutaraldehyde in 0.1 M Sorenson's phosphate buffer, dehydrated through a graded series of ethanol and then critical-point dried (Baltec dryer). Samples were coated with 10nm of gold (olaron coating unit) and viewed on a Tescan Vega LMU SEM operated at 8–10 kV. Images were captured and measured in a blinded fashion.

Protein expression and purification

Chlamydomonas reinhardtii and murine full-length ARL3, *Chlamydomonas reinhardtii* ARL13B 18-278, and human full-length UNC119A were expressed and purified as previously described [3]. Human ARL13B 18-278 was cloned into pET20b with an N-terminal 12×His-tag and purified using the same protocol as ARL3 with the addition of 10% glycerol to the purification buffers and storing the protein in 5% glycerol. p.Arg149His mutations in *ARL3* and p.Glu86Arg mutation in *ARL13B* were introduced using the Q5 site-directed mutagenesis kit (NEB).

Guanine nucleotide exchange assay

Murine ARL3 used for GST pull-downs was exchanged with the non-hydrolysable analogue of GTP, GppNHp. 300 µM ARL3 was incubated with 2 mM GppNHp and 0.14 U/µL Alkaline Phosphatase (Roche) overnight at 15°C. Excess nucleotides were removed by size exclusion chromatography on a Superdex S200 Increase column (GE Healthcare). GppNHp-loaded ARL3 was quantified using HPLC analysis and C18 columns (UltiMate3000, Thermo Fisher Scientific). For the GEF assay, nucleotide exchange of murine and *Chlamydomonas reinhardtii* ARL3 proteins with mantGDP was carried out by incubating 100 µM ARL3 protein with 200 µM mantGDP for 2 h at room temperature in the presence of 50 mM EDTA. For nucleotide exchange to GppNHp on human and *Chlamydomonas reinhardtii* ARL13B, 100 µM ARL13B was incubated with 500 µM GppNHp and 50 mM EDTA, also for 2 h at room pressure. All reactions were carried out in 20 mM tris pH 7.5, 150 mM NaCl, 5 mM $MgCl_2$ and 2 mM DTT and were stopped by adding 100 mM $MgCl_2$. Unbound nucleotides were removed by using a PD10 desalting column (GE Healthcare), and the proteins were concentrated using Amicon Ultra 0.5 mL units (Merck Millipore) with a molecular weight cut-off of 3 kDa.

All reactions were carried out in 20 mM Tris pH 7.5, 150 mM NaCl, 5 mM $MgCl_2$ and 2 mM DTT at room temperature. Fluorescence polarisation was measured at an excitation of 366 nm and emission of 450 nm. 0.5 µM of *Cr*ARL3•mantGDP was measured for 100 s, after which 10 µM GppNHp was added and measured for an additional 100 s. Finally, 5 µM of *Cr*ARL13B•GppNHp was added and changes in fluorescence polarisation were recorded for 300 s. The same was repeated for *Hs*ARL13B, but starting with 1 µM of murine ARL3•mantGDP to which 400 µM GppNHp was added followed by 5 µM *Hs*ARL13B.

GST pull-downs

30 µg of GST-tagged full-length UNC119A and 60 µg of GppNHp-loaded murine ARL3 WT and p.Arg149His were incubated in 20 mM Tris pH 7.5, 150 mM NaCl, 5 mM $MgCl_2$ and 2 mM DTT for 15 minutes at room temperature before adding to Glutathione sepharose 4 FF beads (GE Healthcare) and incubating for a further 20 minutes. Beads were washed 5 times in reaction buffer before eluting with reaction buffer containing 20 mM Glutathione.

Protein Constructs

The following protein constructs were used for protein-protein interaction studies:

Chlamydomonas reinhardtii ARL3^{WT} (C-terminal 6xHis-tag)

MGLLSLIRGL KKKEGEARIL VLGLDNAGKT TILKALSEED ITTITPTQGF NIKSLSRDGF NLKIWDIGGQ KSIRPYWRNY FDQTDALIYV IDSADSKRLS ESEFELTELL QEEKMTGVPL LVFANKQDLV GALAADEIAS TLDLTSIRDR PWQIQACSAK QGTGLKEGME WMMKQVK

Chlamydomonas reinhardtii ARL3^{R148H} (C-terminal 6xHis-tag)

MGLLSLIRGL KKKEGEARIL VLGLDNAGKT TILKALSEED ITTITPTQGF NIKSLSRDGF NLKIWDIGGQ KSIRPYWRNY FDQTDALIYV IDSADSKRLS ESEFELTELL QEEKMTGVPL LVFANKQDLV GALAADEIAS TLDLTSIHDR PWQIQACSAK QGTGLKEGME WMMKQVK

Chlamydomonas reinhardtii ARL13B 18-278 (N-terminal GST-tag)

KITIALLGLD NAGKTTLLNS IQGEVDRDTT PTFGFNSTTL NEGKYKIEVF DLGGGKNIRG VWKKYLAEVH AIVYVVDAAD PGRFEESKMT MAEVLENQFM RDKPICIFAN KQDLPTAAPA AEVVKGLGLA TCRNSHNVFP CTAKMPAGQD VDHRLRDGLK WLVGTVDREF GRLDPRVQTE AEEVRQEEAR KKKEREERLR KQREERLRQQ KEEEERAREV EKENELHDGK APSLLAAGGG VVGAAAAGVN GVMVDEQQEL

Mus musculus ARL3^{WT} (C-terminal 6xHis-tag)

MGLLSILRKL KSAPDQEVRI LLLGLDNAGK TTLLKQLASE DISHITPTQG FNIKSVQSQG FKLNVWDIGG QRKIRPYWRS YFENTDILIY VIDSADRKRF EETGQELTEL LEEEKLSCVP VLIFANKQDL LTAAPASEIA EGLNLHTIRD RVWQIQSCSA LTGEGVQDGM NWVCKNVNAK KK

Mus musculus ARL3^{R149H} (C-terminal 6xHis-tag)

MGLLSILRKL KSAPDQEVRI LLLGLDNAGK TTLLKQLASE DISHITPTQG FNIKSVQSQG FKLNVWDIGG QRKIRPYWRS YFENTDILIY VIDSADRKRF EETGQELTEL LEEEKLSCVP VLIFANKQDL LTAAPASEIA EGLNLHTIHD RVWQIQSCSA LTGEGVQDGM NWVCKNVNAK KK

Homo sapiens ARL13B 18-278 (N-terminal 12xHis-tag)

VRKVTLLMVG LDNAGKTATA KGIQGEYPED VAPTVGFSKI NLRQGKFEVT IFDLGGGIRI RGIWKNYYAE SYGVIFVVDS SDEERMEETK EAMSEMLRHP RISGKPILVL ANKQDKEGAL

GEADVIECLS LEKLVNEHKC LCQIEPCSAI SGYGKKIDKS IKKGLYWLLH VIARDFDALN ERIQKETTEQ RALEEQEKQE RAERVRKLRE ERKQNEQEQA ELDGTSGLAE LDPEPTNPFQ PIASVIIENE GKLEREKKNQ

References

1. Soding, J. (2005). Protein homology detection by HMM-HMM comparison. Bioinformatics 21, 951-960.
2. Sali, A., and Blundell, T.L. (1993). Comparative protein modelling by satisfaction of spatial restraints. Journal of molecular biology 234, 779-815.
3. Ismail, S.A., Chen, Y.X., Miertzschke, M., Vetter, I.R., Koerner, C., and Wittinghofer, A. (2012). Structural basis for Arl3-specific release of myristoylated ciliary cargo from UNC119. The EMBO journal 31, 4085-4094.
