# Supplementary material for: ARL3 Mutations Cause Joubert Syndrome by Disrupting Ciliary Protein Composition
Source: Am J Hum Genet. 2018 Sep 27;103(4):612–20. doi: 10.1016/j.ajhg.2018.08.015 (PMC6174286; doi:10.1016/j.ajhg.2018.08.015)
Supplement: Document S2. Article plus Supplemental Data [file mmc2.pdf]

REPORT

ARL3 Mutations Cause Joubert Syndrome by Disrupting Ciliary Protein Composition

Sumaya Alkanderi,[1,10] Elisa Molinari,[1,10] Ranad Shaheen,[2,10] Yasmin Elmaghloob,[3] Louise A. Stephen,[3] Veronica Sammut,[1] Simon A. Ramsbottom,[1] Shalabh Srivastava,[1,4] George Cairns,[1] Noel Edwards,[1] Sarah J. Rice,[1] Nour Ewida,[2] Amal Alhashem,[5,6] Kathryn White,[7] Colin G. Miles,[1] David H. Steel,[1,8] Fowzan S. Alkuraya,[2,6,11] Shehab Ismail,[3,9,11,*] and John A. Sayer[1,4,11,*]

Joubert syndrome (JBTS) is a genetically heterogeneous autosomal-recessive neurodevelopmental ciliopathy. We investigated further the underlying genetic etiology of Joubert syndrome by studying two unrelated families in whom JBTS was not associated with pathogenic variants in known JBTS-associated genes. Combined autozygosity mapping of both families highlighted a candidate locus on chromosome 10 (chr10: 101569997–109106128, UCSC Genome Browser hg 19), and exome sequencing revealed two missense variants in *ARL3* within the candidate locus. The encoded protein, ADP ribosylation factor-like GTPase 3 (ARL3), is a small GTP-binding protein that is involved in directing lipid-modified proteins into the cilium in a GTP-dependent manner. Both missense variants replace the highly conserved Arg149 residue, which we show to be necessary for the interaction with its guanine nucleotide exchange factor ARL13B, such that the mutant protein is associated with reduced INPP5E and NPHP3 localization in cilia. We propose that ARL3 provides a potential hub in the network of proteins implicated in ciliopathies, whereby perturbation of ARL3 leads to the mislocalization of multiple ciliary proteins as a result of abnormal displacement of lipidated protein cargo.

Mutations in genes that are involved in the structure or function of the primary cilium give rise to a range of disorders known as ciliopathies.[1] These are typically multi-system disorders, as seen in the archetypal ciliopathy Joubert syndrome (JBTS), which is characterized clinically by brain malformations that result in developmental delay, oculomotor apraxia, and hypotonia.[2] In addition to the neurodevelopmental phenotype, retinal and renal diseases are often associated with JBTS.[3] Now more than 35 genes are known to cause JBTS when mutated in an autosomal-recessive or X-linked manner[4–7] (also see GeneReviews in Web Resources). Genetic approaches have moved from traditional linkage studies and homozygosity mapping to exome sequencing strategies, protein interaction networks,[8] and genome-wide small interfering RNA screens,[9] allowing a rapid rate of gene discovery. Despite these advancements, which made it possible for the majority of JBTS cases to have a genetic diagnosis,[10] many cases of JBTS remain genetically unsolved, and critically, the inter-relationships between the proteins encoded by these genes and the underlying disease mechanisms remain poorly understood. Here, we used a combination of autozygosity mapping and whole-exome sequencing (WES)[11,12] in two unsolved JBTS-affected families and identified likely deleterious variants in *ARL3* (MIM: 60495). We further investigate the mechanistic impact of this mutation and show that the mutant ARL3 is irresponsive to the guanine nucleotide exchange factor (GEF) activity of ARL13B and causes associated defects in ciliary proteins in affected individuals' fibroblasts.

Family 1 is a Saudi Arabian family comprising first-cousin healthy parents and six children, including the 5-year-old male index individual (II:5; Figure 1). His clinical features include developmental delay, multicystic dysplastic left kidney, night blindness, and mild dysmorphic features, including ptosis (Figure 1 and Table 1). Magnetic resonance imaging (MRI) of the brain showed severe vermis hypoplasia with abnormal thick cerebellar peduncles configured in the shape of a typical molar tooth sign (Figure 1B), as well as abnormal configuration of the midbrain, thinning of the pontomesencephalic junction and midportion of the midbrain, and mild decreased brain volume with a paucity of white matter in the frontotemporal region and dilated ventricular system. This family is part of a large ciliopathy cohort (enrolled in a research protocol approved by King Faisal Specialist Hospital and Research Center research advisory council 2080006 after providing informed consent). Family 2, originating from Pakistan, is also consanguineous and comprises three affected children with a clinical syndrome in keeping with JBTS (II;1, II:4, and II:5; Table 1 and Figure 1). The eldest sibling (II:1) presented with hypotonia and psychomotor delay. Subsequently, the child developed night blindness and bilateral visual loss by 4 years of age. She

[1]Institute of Genetic Medicine, Newcastle University, International Centre for Life, Central Parkway, Newcastle upon Tyne NE1 3BZ, UK; [2]Department of Genetics, King Faisal Specialist Hospital and Research Center, Riyadh 11211, Saudi Arabia; [3]Structural Biology of the Cilia Lab, Beatson Institute for Cancer Research, Switchback Road, Bearsden, Glasgow G61 1BD, UK; [4]Renal Services, Newcastle Hospitals NHS Foundation Trust, Newcastle upon Tyne NE7 7DN, UK; [5]Department of Pediatrics, Prince Sultan Medical Military City, Riyadh 12233, Saudi Arabia; [6]Department of Anatomy and Cell Biology, College of Medicine, Alfaisal University, Riyadh 11533, Saudi Arabia; [7]Electron Microscopy Research Services, Newcastle University, Newcastle upon Tyne NE2 4HH, UK; [8]Sunderland Eye Infirmary, Queen Alexandra Road, Sunderland SR2 9HP, UK; [9]Institute of Cancer Sciences, University of Glasgow, Glasgow G61 1QH, UK
[10]These authors contributed equally to this work
[11]These authors contributed equally to this work
*Correspondence: s.ismail@beatson.gla.ac.uk (S.I.), john.sayer@ncl.ac.uk (J.A.S.)
https://doi.org/10.1016/j.ajhg.2018.08.015.
© 2018 The Author(s). This is an open access article under the CC BY license (http://creativecommons.org/licenses/by/4.0/).

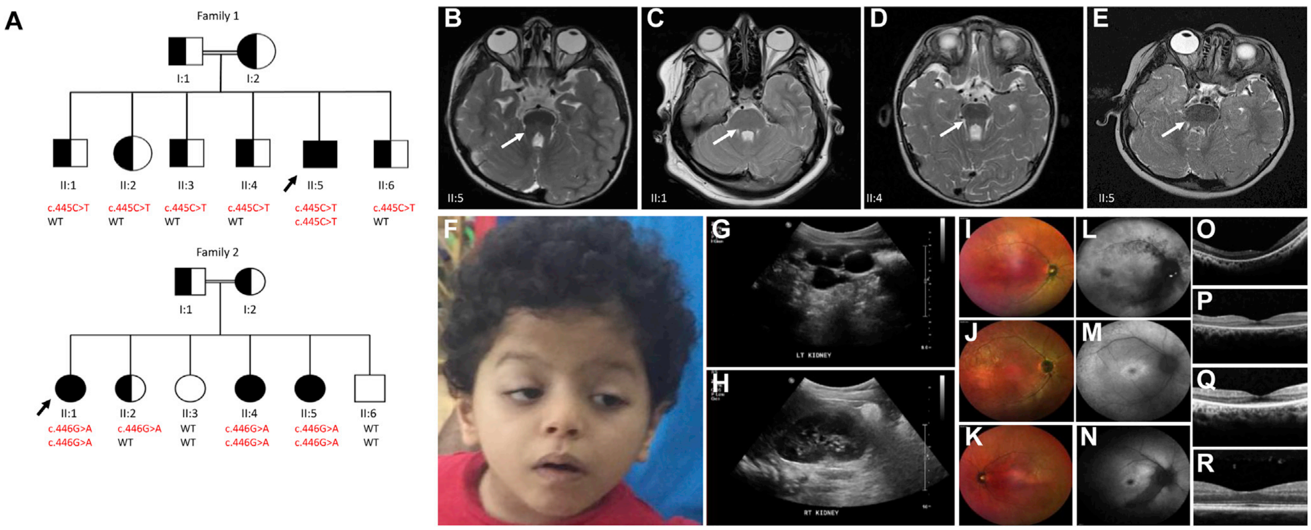

Figure 1. Clinical and Radiological Images of the Affected Members of the Two Families Included in This Study

(A) A pedigree of the two families shows the number of affected siblings in each family and the outcome of segregation analysis (affected, shaded; carriers, half-shaded; and WT, unshaded). The proband in each family is indicated by a black arrow. Genotypes for the proband and their siblings are shown.

(B–E) Brain MRI of the four affected individuals (B, II:5 in family 1; C, D, and E, II:1, II:4, and II:5 in family 2) in this study shows evidence of a molar tooth sign, cerebellar vermis hypoplasia, and elongation of the superior cerebellar peduncles (arrowed).

(F) Facial photo of the proband (II:5) in family 1 shows dysmorphic features (depressed nasal bridge, upturned nares, ptosis, arched eyebrows, synophrys, telecanthus, and low-set ears).

(G and H) Ultrasound scan image of the kidneys of the affected member in family 1 (II:5) shows an echogenic left multicystic dysplastic kidney (G) and an unaffected right kidney (H).

(I–R) Retinal imaging, including multicolor scanning laser fundal images of the eyes, of the three affected siblings in family 2 (II:1, II:4, and II:5) shows granular alterations of the retinal pigment epithelium and subtle spicule formation, particularly around the major vascular arcades, and arteriolar attenuation (I, II:1; J, II:4; K, II:5). Autofluorescence images show stippled hypo-autofluorescence areas concentrated around the arcades (L, II:1) and hyper-autofluorescence around fovea (M, II:4; N, II:5). Horizontal optical coherence tomography scans demonstrate thinning of the outer nuclear layer and loss of ellipsoid and external limiting membrane lines with preservation of inner retinal lamination in all three siblings (O, II:1; P, II:4; Q, II:5). A horizontal optical coherence tomography scan of a healthy control individual is shown for comparison (R).

also had recurrent urinary-tract infections (Table 1). Clinical investigations revealed the molar tooth sign that is typical of JBTS on brain MRI, as well as retinal dystrophy (Figure 1). The other two affected siblings (II:4 and II:5) had very similar presentations with predominating brain and retinal features (Table 1 and Figure 1). Siblings II:1 and II:4 experienced problems with thermoregulation, which implies brainstem involvement, as well as the known cerebellar defects typical of JBTS. This family was enrolled in a research protocol approved by the National Research Ethics Service (09/H0903/36) after providing informed consent.

Exome sequencing of the index individual in each family and variant filtering were performed as previously described.[7] In brief, WES was performed with the TruSeq Exome Enrichment Kit from Illumina. Coding and splicing homozygous variants were considered as candidates only if they were present within the candidate locus, had a frequency < 0.1% in publicly available variant databases (1000 Genomes, NHLBI Exome Sequencing Project Exome Variant Server, and Genome Aggregation Database [gnomAD]) and a database of in-house ethnically matched exomes (Saudi Human Genome Program; totaling 2,379 exomes), and were predicted to be pathogenic *in silico*.

Interestingly, both families were flagged by the corresponding research group because exome sequencing did not reveal a likely deleterious bi-allelic variant in any of the established JBTS-related genes. Through an investigator-initiated collaboration, an attempt was made to exploit the consanguineous nature of both families, which can readily reveal a potentially unifying etiology if they have an overlapping autozygome, as previously described.[7] In brief, we performed genome-wide genotyping with the Axiom SNP Chip platform from Affymetrix and the Sure Select V4 platform from Agilent Technologies and then determined autozygomes by using HomozygosityMapper on all available family members. This revealed a single critical locus (chr10: 101,569,997–109,106,128, UCSC Genome Browser hg 19) (Figure 2A). This locus spans 57 genes, none of which is known to be linked to a ciliopathy phenotype. After re-analyzing the exome variants by only considering variants within this locus (Tables S1 and S2), we found a single previously unreported variant in *ARL3* in each index individual: c.445C>T (p.Arg149Cys) (GenBank: NM_004311.3) in family 1 and c.446G>A (p.Arg149His) (GenBank: NM_004311.3) in family 2 (Figure 2B). Both homozygous variants fully co-segregated with the JBTS phenotype in each family.

Table 1. Clinical Features of JBTS in Affected Family Members

	Family 1	Family 2		
	II:5	II:1	II:4	II:5
Age (years)	5	21	12	9
Central nervous symptoms	developmental delay, ataxia	developmental delay, ataxia	developmental delay, ataxia	developmental delay, ataxia
Ocular symptoms	ptosis, rod-cone dystrophy, night blindness, bilateral visual pathway involvement	rod-cone dystrophy, night blindness, progressive visual loss	rod-cone dystrophy, night blindness, progressive visual loss	rod-cone dystrophy, night blindness, progressive visual loss, oculomotor apraxia
eGFR (mL/min/1.73 m^2)	NA	75	>90	>90
Renal symptoms	none	recurrent UTI	none	recurrent UTI
USS renal	left multicystic dysplastic kidney, right grade I hydronephrosis	bilateral renal scarring	normal USS	unequal kidney size
Other	single palmar crease, pectus carinatum, normal ABR	thermoregulation problems, episode of transverse myelitis	thermoregulation problems, sleep apnea	none

Abbreviations are as follows: ABR, auditory brainstem response; eGFR, estimated glomerular filtration rate; NA, not available; USS, ultrasound scan; and UTI, urinary-tract infection.

ARL3 is a highly conserved gene, and its encoded protein, the small G-protein ARL3, localizes to the cilium and is crucial for ciliogenesis and axoneme formation, as well as cargo displacement of lipidated proteins in the cilium.[13] *ARL3* variants have also been reported in association with retinal dystrophy.[14] Among ARL3 effectors are the GDI-like solubilizing factors (GSFs) PDE6D, UNC119A, and UNC119B, whose interactions are guanosine triphosphate (GTP) dependent. GSFs bind to and solubilize prenylated and myristoylated proteins, which are released by ARL3-GTP acting as an allosteric release factor.[15,16] The cilia-specific protein ARL13B acts as a specific GEF for ARL3, whereas retinitis protein 2 (RP2) functions as an ARL3 GTPase-activating protein (GAP) and is localized in the pre-ciliary compartment.[17,18] This segregation of a GEF and a GAP is proposed to create an ARL3-GTP gradient inside the cilium,[19] which ensures the destination-specific release of lipid-modified ciliary proteins, solubilized by GSFs.[20]

The ARL3 Arg149 residue is highly conserved throughout evolution (Figure 2C), and *in silico* prediction tools suggest that either missense change is likely to be pathogenic (Table S3). Homology models of ARL3 reveal that the two variants, which are located in a loop between the α4 and β6 domains (Figure 3A), are predicted to disrupt the interaction of ARL13B with ARL3 because it requires this precise residue (Arg149) for its interaction (Figure 3B). Superimposing all known structures of ARL3 in complex with its effectors, GAP and GEFS, the ARL3 Arg149 residue is exclusively present in the interface between ARL3 and ARL13B and is involved in an ionic interaction with the conserved ARL13B Glu88 residue (Figure 3B). To functionally investigate the effect of the mutation on the interaction with ARL13B, we performed a GEF fluorescence-based polarization experiment.[19] Wild-type (WT) and mutant p.Arg149His versions of murine ARL3 (98.35% sequence identity to human ARL3) were bound to fluorescently labeled GDP, and an excess of unlabeled GTP was added in the presence or absence of human ARL13B. We then followed the capability for nucleotide exchange of both versions of the protein by recording the fluorescence polarization over time. Upon addition of the ARL13B GEF, WT ARL3 showed a clear acceleration of nucleotide exchange. Under similar conditions, mutant p.Arg149His ARL3 failed to show acceleration of nucleotide exchange in the presence of ARL13B (Figure 3C). The integrity of the mutant protein was confirmed by pull-down, whereby both WT and p.Arg149His ARL3 proteins were pulled down equally by UNC119A (Figure 3F and Figure S1). Furthermore, we confirmed our results by using the highly conserved *C. reinhardtii* ARL3 (WT and mutant p.Arg148His) and ARL13B (Figure 3D). To further investigate the importance of the ARL3-ARL13B interaction, we carried out the reverse charge variant p.Glu86Arg in ARL13B by using *C. reinhardtii* proteins. As expected, p.Glu86Arg ARL13B was not able to accelerate the nucleotide exchange of WT ARL3 (Figure 3E). From these experiments, we conclude that p.Arg149His ARL3 disrupts the interaction with ARL13B and is defective in ARL13B-assisted nucleotide exchange.

To determine ciliary morphology, we obtained fibroblasts from all three affected individuals in family 2 (II:1, II:4, and II:5) plus control individuals (both parents [I:1 and I:2] and an unaffected sibling [II:3]). Primary cilia identified by ARL13B antibodies were of normal length in affected individuals (mean length = 5.9, 7.8, and 6.8 μm in II:1, II:4, and II:5, respectively) and control individuals (mean length = 5.7 and 6.0 μm in the parents and 6.1 μm in the unaffected sibling), and there were no

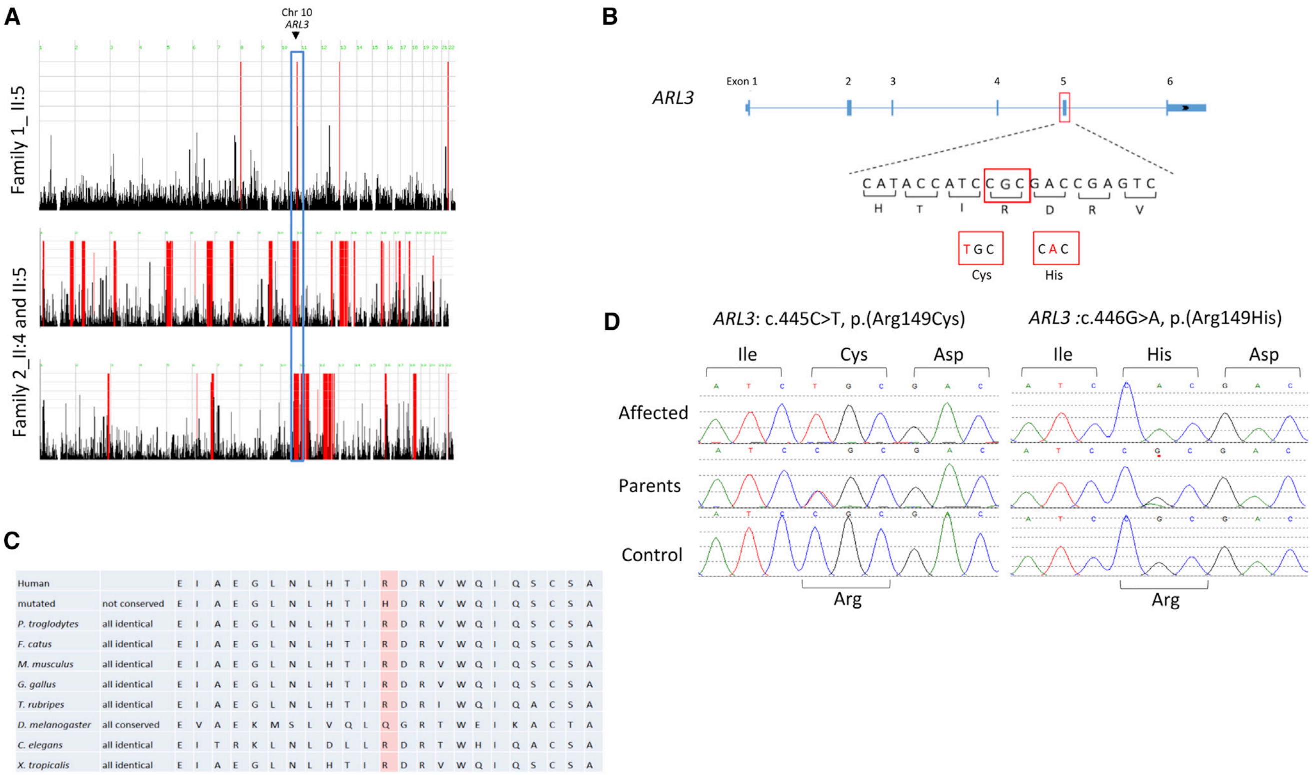

Figure 2. Molecular Genetic Investigations of the Two JBTS-Affected Families

(A) Genome-wide homozygosity mapping shows the shared homozygous region between the affected members of the two families on chromosome 10 (blue rectangle). Regions of homozygosity are shown in red, and the position of *ARL3* is marked with a black arrow.
(B) Schematic representation to *ARL3* with the homozygous missense variants located in exon 5.
(C) Evolutionary conservation of residue Arg149, which is highly conserved throughout all species shown except *D. melanogaster*.
(D) Sequence chromatograms of the two different *ARL3* variants described in this study.

significant differences between the two groups (Figure S2). There was also no difference in the percentage of ciliation rates between affected and control fibroblasts (Figure S2). Scanning electron microscopy confirmed these findings of no significant changes in cilia length or structural appearance (Figure S3).

ARL3 functions as an allosteric release factor of all GSFs members: PDE6D, UNC119A, and UNC119B. Whereas PDE6D is involved in the trafficking of prenylated proteins, UNC119A and UNC119B traffic myristoylated proteins.[19] Given that ARL3 exerts its releasing function only when bound to GTP, we expected the ciliary localization of the GSF cargo to be impaired. The INPP5E, GRK1, and PDE6 catalytic subunits are among the prenylated GSF ciliary cargo,[20] whereas the myristoylated ciliary cargo includes NPHP3, GNAT1, and Cystin1.[22] To test our hypothesis, we examined cilia for protein content of both the prenylated INPP5E and myristoylated NPHP3. *ARL3*-mutant cilia demonstrated a significant loss of both INPP5E and NPHP3 content (Figure 4 and Figures S4–S6), indicating that WT ARL3 is required for normal release of these cargos into the ciliary axoneme. To confirm these phenotypes as specific to the loss of ARL3 function, we sought to determine the ciliary content of GLI3 in WT and ARL3-mutant cilia. GLI3 translocation is independent of GSF transport and relies upon intraflagellar transport proteins and Sonic Hedgehog signal transduction.[23] Consistent with morphologically normal cilia in *ARL3*-mutant fibroblasts, no defect in ciliary GLI3 was observed after stimulation with SAG, a Hedgehog pathway agonist. The amounts of total ciliary GLI3 and ciliary tip GLI3 were unchanged between affected and control individuals (Figure S7), confirming that the ciliary Hedgehog signaling pathway is not disturbed by this particular *ARL3* mutation. Together, these data substantiate a role for ARL3 in the release of both prenylated and myristoylated ciliary cargo, which is disrupted by the p.Arg149His ARL3 variant.

We present *ARL3* as a ciliopathy- and JBTS-associated gene. *Ld*ARL-3A, a *Leishmania* homolog of ARL3, is an essential component of flagellum formation.[24] *Arl3* knockdown has previously been investigated in a gene-trap murine model, where *Arl3* was disrupted after the first exon.[25] These $Arl3^{-/-}$ mice, which represent a null allele, developed a severe ciliopathy phenotype with pronounced cystic kidney disease, pancreatic hypoplasia, ductal plate malformation within the liver, and retinal dystrophy with impaired photoreceptor development.[25] The mice died within 3 weeks of age, indicating a severe phenotype, which is much more detrimental than that of our human subjects, who carry a missense mutation. We speculate

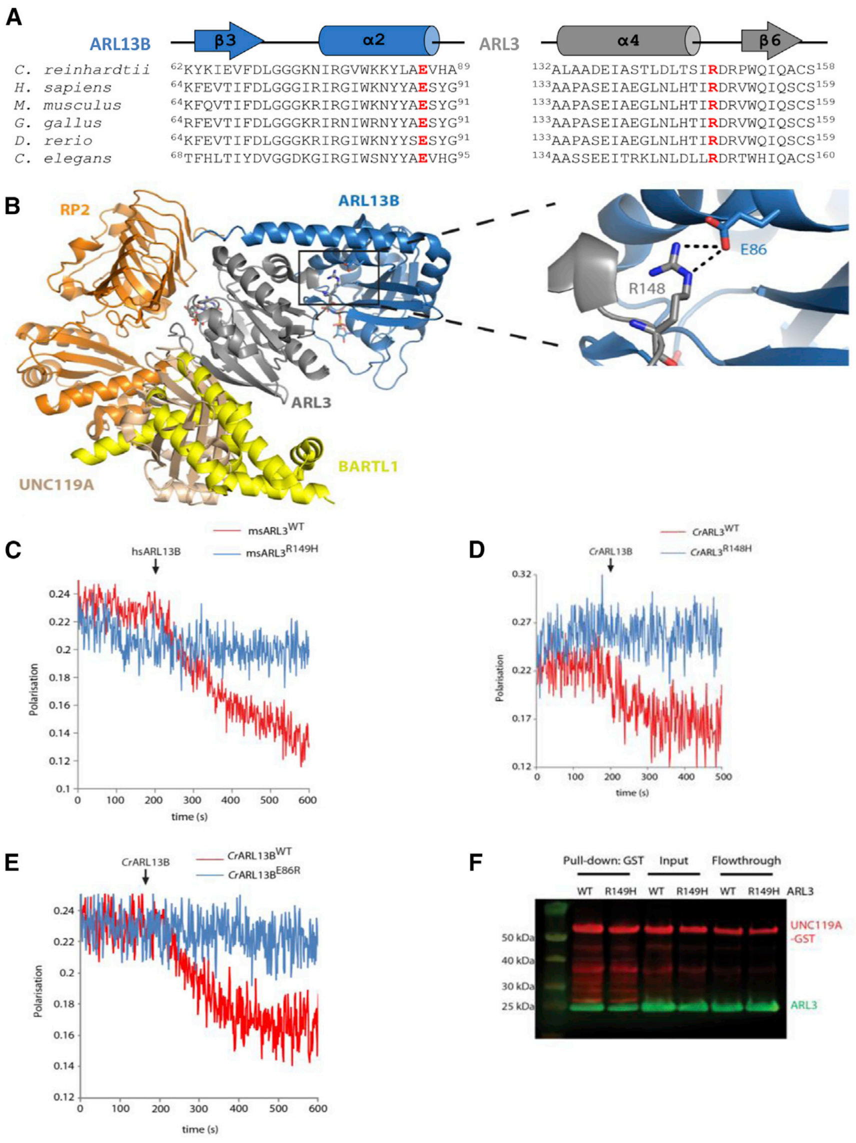

Figure 3. The Human ARL13B-ARL3 Complex Is Predicted to Involve an Interaction between Evolutionarily Conserved Glutamate and Arginine Residues

(A) Partial amino acid sequence alignments of the ciliary GEF, ARL13B, and ARL3. Highlighted in red are the evolutionarily conserved glutamate residue located in the switch II domain of ARL13B (E86 [Glu86] in *C. reinhardtii* ARL13B [*Cr*ARL13B]) and the arginine residue in the loop region between the α4 and β6 domains of ARL3 (R148 [Arg148] in *Cr*ARL3).
(B) Superimposition of the crystal structures of ARL3 (gray) in complex with its known interactors: the effectors UNC119A (salmon; PDB: 4GOJ[15]) and BARTL1 (yellow; PDB: 4ZI2[21]), the GAP RP2 (orange; PDB: 3BH6[17]), and GEF ARL13B (blue; PDB: 5DI3[19]). On the right side is a zoomed-in view of the salt bridge between Glu86 and Arg148 at the *Cr*ARL13B-*Cr*ARL3 complex interface.
(C) Assay of GEF activity for murine WT ARL3 ($ARL3^{WT}$) and p.Arg149His ARL3 ($ARL3^{R149H}$). Fluorescence polarization was measured for 1 μM mantGDP-loaded ARL3, to which 400 μM GppNHp and 5 μM *H. sapiens* ARL13B (*Hs*ARL13B) were added. Nucleotide exchange was shown by only $ARL3^{WT}$.
(D) Assay of GEF activity with fluorescence polarization measurements of 0.5 μM mantGDP-loaded *Cr*$ARL3^{WT}$ and *Cr*$ARL3^{R148H}$, to which 10 μM GppNHp (GTP analog) and 5 μM *Cr*ARL13B·GppNHp were added at the indicated time points. Only *Cr*$ARL3^{WT}$ showed nucleotide exchange, as indicated by the drop in fluorescence polarization.
(E) Assay of GEF activity with fluorescence polarization measurements of 0.5 μM mantGDP-loaded *Cr*$ARL3^{WT}$ and 5 μM *Cr*ARL13B·$GppNHp^{WT}$ or *Cr*$Arl13b^{E86R}$, to which 10 μM GppNHp (GTP analog) was added at the indicated time points. Only *Cr*$Arl13b^{WT}$ showed nucleotide exchange, as indicated by the drop in fluorescence polarization.
(F) 30 μg of full-length UNC119A-GST was used to pull down 60 μg of murine $ARL3^{WT}$ and $ARL3^{R149H}$ that were loaded with the GTP analog GppNHp. Proteins were detected on immunoblots with anti-GST (red) and anti-His (green) antibodies.

that nonsense mutations in *ARL3* in humans could cause more pronounced ciliopathy phenotypes, such as the perinatally lethal ciliopathy Meckel syndrome,[26] and could go some way to explaining why such a fundamental gene has previously not been identified in ciliopathy syndromes. It is noteworthy that the ExAC Browser and gnomAD do not have any homozygous pathogenic variants reported within *ARL3* and that the gene is relatively intolerant to variation (positive *Z* score of 0.44). We did not identify any additional *ARL3* pathogenic variants in our WES databases, which are relatively enriched with autozygosity, or in a cohort of 35 unsolved JBTS-affected individuals.

In humans, Strom et al. previously reported the heterozygous missense variant c.269A>G (p.Tyr90Cys) in *ARL3* in a European-descent pedigree with non-syndromic retinitis pigmentosa.[27] The variant, which was rare, appeared *de novo* and was predicted to be pathogenic, was confirmed as heterozygous in three affected individuals, and was transmitted in an autosomal-dominant fashion. A second allele was not identified, and mechanistic evaluation was not carried out. On the other hand, here we have identified bi-allelic *ARL3* changes that fully segregate with a classical JBTS phenotype, including retinal changes. Thus, although the connection between the *de novo ARL3* variant and retinitis pigmentosa remains unexplained, it seems that bi-allelic *ARL3* deleterious variants are sufficient to cause JBTS. The involvement of ciliopathy-associated genes in non-syndromic retinitis pigmentosa has been well described, so it would be of interest for the affected individual reported by Strom et al. to be investigated for the possibility of a second deleterious allele in *trans* in *ARL3*. It is also possible that, as reported here, bi-allelic mutations

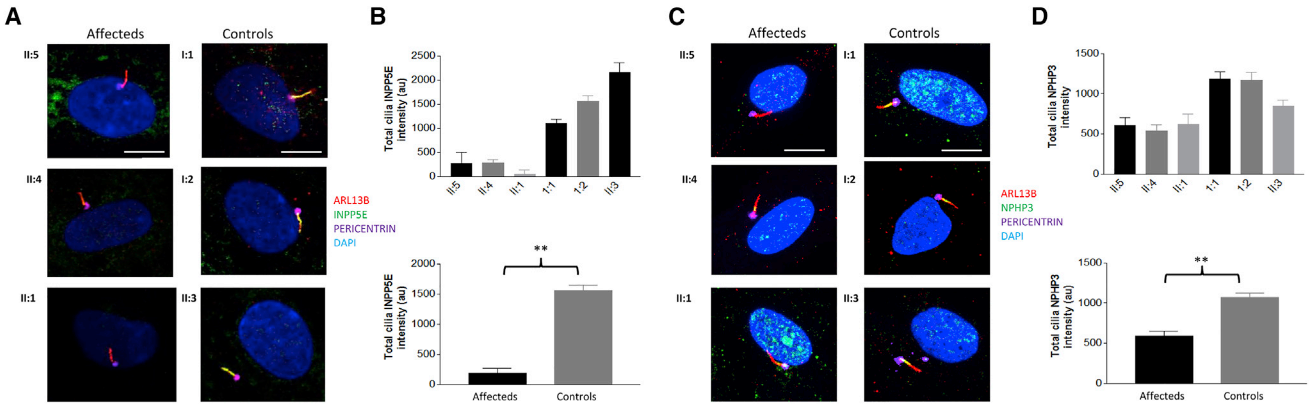

Figure 4. Characterization of Ciliary Phenotype in *ARL3*-Mutant Fibroblasts from Family 2
(A and C) Affected and control fibroblasts were observed under high-power immunofluorescence for determining ciliary expression of (A) INPP5E and (C) NPHP3. Cilia were localized with anti-ARL13B (red) and anti-PERICENTRIN (magenta) for the identification of the ciliary membrane and the base of cilia, respectively. Scale bars, 10 μm.
(B) Quantification of ciliary localization of INPP5E (**$p < 0.0001$, unpaired t test, $n > 150$ cilia for each group). Total cilia INPP5E in control fibroblast (II:3) cilia is higher than in heterozygous fibroblast (I:1 and II:2) cilia.
(D) Quantification of ciliary localization of NPHP3 (**$p < 0.0001$, unpaired t test, $n > 150$ cilia for each group).

in *ARL3* give rise to an extended phenotype compared with its reported dominant phenotype. A growing number of genes are known to cause distinct phenotypes according to whether dominant or recessive variants are inherited. For retinitis pigmentosa, mutations (typically nonsense) in *RP1* were initially described in an autosomal-dominant pattern,[28] followed by autosomal-recessive (homozygous missense) variants.[29] For Gillespie syndrome, a form of non-progressive cerebellar ataxia, both bi-allelic and mono-allelic mutations in *ITPR1* (MIM: 147265) have been reported,[30] and the single heterozygous mutations were thought to exert a dominant-negative effect. In addition, variants in genes known only to be related to autosomal-dominant disease have been found in association with recessive mutations, both where the phenotypes are similar but more severe (*ACTG2*-related visceral myopathy [MIM: 102545]) and where distinctly different phenotypes have been observed (*FBN2*-related myopathy [MIM: 612570] and *CSF1R*-related brain malformation [MIM: 164770]).[31]

Interestingly, pathogenic variants in the ARL3 interaction partners ARL13B and PDE6D also cause JBTS. *ARL13B* (MIM: 608922) mutations were reported in individuals with a classical neurodevelopmental JBTS phenotype (JBTS8) without prominent renal phenotypes.[32] It is particularly noteworthy that some affected individuals had a small occipital encephalocele, indicating that more severe brain phenotypes could be likely. *PDE6D* (MIM: 602676) mutations have been reported in three siblings with JBTS (JBTS22) and associated retinal and post-axial polydactyly phenotypes, as well as kidney hypoplasia.[33] Furthermore, the disrupted ciliary cargo proteins (INPP5E and NPHP3) we identified are also responsible for JBTS phenotypes when their encoding genes are mutated. *INPP5E* (MIM: 613037) mutations cause JBTS1 and were identified in a cohort of JBTS-affected individuals with mainly neurological features and some retinopathy but without kidney disease or polydactyly,[34] suggesting a lack of Hedgehog signaling defects.[35,36] In contrast, *NPHP3* (MIM: 608002) mutations were initially identified as causing an adolescent form of nephronophthisis, a progressive form of renal failure.[37] Since this initial report, *NPHP3* mutations have been associated with a wider spectrum of disease, including infantile nephronophthisis (resulting in end-stage renal failure before 5 years of age[38]) and Meckel syndrome.[39] The full spectrum of disease phenotypes secondary to *ARL3* mutation therefore remains to be determined, but *ARL3* is widely expressed and fundamental to the ciliary localization of a wide range of proteins. Therefore, one could predict that any severity of JBTS disease with retinal and renal involvement is possible. It will be important to study the tissue-specific roles of *ARL3* and the implications of disrupting expression in these tissues.

The primary cilium exerts its function by concentrating certain proteins and lipids, thereby maintaining a distinct composition and function. ARL13B is specifically localized in the cilium, creating a high ciliary concentration of ARL3-GTP, which in turn produces a hotspot for releasing GSF-bound cargo. Our study underscores the physiological importance of this mechanism because the human mutation we characterize, c.446G>A (p.Arg149His), disrupts the interaction between ARL13B and ARL3 and results in loss of GSF cargo concentration in the cilia (Figure 5). As we have described, ciliopathies such as JBTS show overlapping phenotypes, and one gene can be involved in a broad range of ciliopathy phenotypes. A cause of this overlap is most likely the fact that proteins do not work in isolation but in networks. Indeed, it has been shown that ciliopathy-associated proteins form different modules that cross talk and interact together.[8,40] Through the identification of *ARL3* variants as a cause of JBTS, we show that ARL3

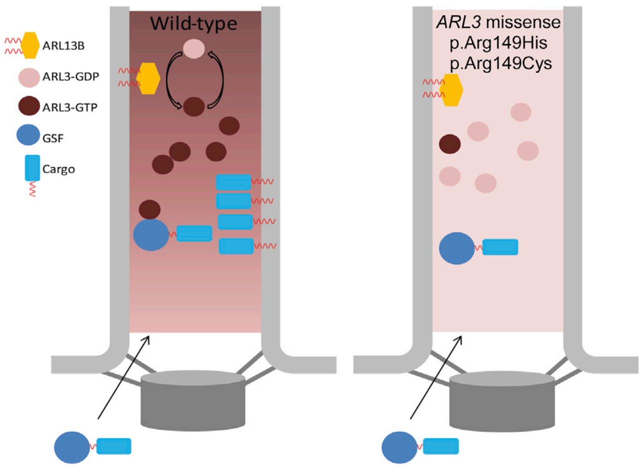

Figure 5. Model of GSF-Cargo Release in Cilia with WT ARL3 versus p.Arg149 ARL3 Missense Variants
ARL13B assists ARL3 in cilia to exchange its bound GDP to GTP. The specific localization of ARL13B in the cilia creates a high concentration of ARL3-GTP. ARL3-GTP in turn can release the cargo bound to its cognate GSF, resulting in ciliary localization. Missense variants of ARL3 (including p.Arg149His and p.Arg149Cys) are not able to interact with ARL13B, and the ARL3GTP concentration is therefore low in the cilia, resulting in inefficient release of GSF cargo in the cilia.

provides a hub within the network of ciliopathy-associated genes, whereby perturbation of ARL3 results in the mislocalization of multiple ciliary proteins, including INPP5E and NPHP3. Our mechanistic model might provide good starting points for therapeutic intervention where small molecules can be used to release GSF-bound cargo and compensate for the loss of ARL3 release activity. Directing such therapies to the kidney in individuals with JBTS-associated renal dysfunction and to the retina in cases of progressive visual loss secondary to JBTS would be desirable. Using small molecules to disrupt GSF-cargo interaction has been reported,[41,42] and it will be important to test those small molecules with regard to cilia function and their application in ciliopathies. Nevertheless, a challenge will be to target these small molecules specifically to the cilia, where the function of ARL3 is concentrated, to assure the correct targeting of ciliary GSF cargo.

In conclusion, we have identified *ARL3* missense variants as a likely cause of JBTS. On the basis of limited observations, the phenotype related to variants in this gene seem to be a cerebello-retinal presentation similar to that caused, for instance, by pathogenic variants in *AHI1* (MIM: 608894).[43,44] In fact, none of the affected individuals presented with any striking additional features, and renal involvement was inconstant. Because effective treatments for JBTS are lacking at present, genotype-phenotype correlations could prove useful in giving prognostic indications to families. We have shown that substitution of arginine at position 149 disrupts the known interaction between ARL3 and ARL13B and thus prevents the correct release of intra-ciliary cargos, including INPP5E and NPHP3. Furthermore, we propose that therapeutic manipulation of ciliary cargo release could provide an innovative treatment mechanism for human ciliopathies such as JBTS.

Accession Numbers

Gene variants reported in this manuscript (for subjects 00179519, 00179520, 00179521, and 00179522) have been submitted to LOVD at https://databases.lovd.nl/shared/genes/ARL3.

Supplemental Data

Supplemental Data include Supplemental Material and Methods (including homology modeling and protein constructs), seven figures, and three tables and can be found with this article online at https://doi.org/10.1016/j.ajhg.2018.08.015.

Acknowledgments

This work was funded by the Medical Research Council (MR/M012212/1), Kidney Research UK (post-doctoral fellowship PDF_003_20151124 to S.A.R.), King Salman Center for Disability Research (F.S.A.), King Abdulaziz City for Science and Technology (13-BIO1113-20 to F.S.A.), Saudi Human Genome Program (F.S.A.), Northern Counties Kidney Research Fund (J.A.S. and E.M.), and Cancer Research UK (core funding award A19257 to Y.E., L.S., and S.I.).

Declaration of Interests

The authors declare no competing interests.

Received: June 12, 2018
Accepted: August 24, 2018
Published: September 27, 2018

Web Resources

Combined Annotation Dependent Depletion (CADD), http://cadd.gs.washington.edu/
ExAC Browser, http://exac.broadinstitute.org/
GenBank, https://www.ncbi.nlm.nih.gov/genbank/
GeneReviews, Parisi, M., and Glass, I. (1993). Joubert Syndrome, https://www.ncbi.nlm.nih.gov/books/NBK1325/
Genome Aggregation Database (gnomAD), http://gnomad.broadinstitute.org/
HomozygosityMapper, http://www.homozygositymapper.org/
OMIM, https://www.omim.org/
Leiden Open Variation Database (LOVD), https://databases.lovd.nl/shared/genes/ARL3
MutationTaster, http://www.mutationtaster.org/
RSCB Protein Data Bank, https://www.rcsb.org/pdb/home/home.do
UCSC Genome Browser, https://genome.ucsc.edu/

References

1. Hildebrandt, F., Benzing, T., and Katsanis, N. (2011). Ciliopathies. N. Engl. J. Med. *364*, 1533–1543.
2. Romani, M., Micalizzi, A., and Valente, E.M. (2013). Joubert syndrome: congenital cerebellar ataxia with the molar tooth. Lancet Neurol. *12*, 894–905.

3. Srivastava, S., Ramsbottom, S.A., Molinari, E., Alkanderi, S., Filby, A., White, K., Henry, C., Saunier, S., Miles, C.G., and Sayer, J.A. (2017). A human patient-derived cellular model of Joubert syndrome reveals ciliary defects which can be rescued with targeted therapies. Hum. Mol. Genet. *26*, 4657–4667.
4. Akizu, N., Silhavy, J.L., Rosti, R.O., Scott, E., Fenstermaker, A.G., Schroth, J., Zaki, M.S., Sanchez, H., Gupta, N., Kabra, M., et al. (2014). Mutations in *CSPP1* lead to classical Joubert syndrome. Am. J. Hum. Genet. *94*, 80–86.
5. Romani, M., Micalizzi, A., Kraoua, I., Dotti, M.T., Cavallin, M., Sztriha, L., Ruta, R., Mancini, F., Mazza, T., Castellana, S., et al. (2014). Mutations in *B9D1* and *MKS1* cause mild Joubert syndrome: expanding the genetic overlap with the lethal ciliopathy Meckel syndrome. Orphanet J. Rare Dis. *9*, 72.
6. Roosing, S., Romani, M., Isrie, M., Rosti, R.O., Micalizzi, A., Musaev, D., Mazza, T., Al-Gazali, L., Altunoglu, U., Boltshauser, E., et al. (2016). Mutations in *CEP120* cause Joubert syndrome as well as complex ciliopathy phenotypes. J. Med. Genet. *53*, 608–615.
7. Van De Weghe, J.C., Rusterholz, T.D.S., Latour, B., Grout, M.E., Aldinger, K.A., Shaheen, R., Dempsey, J.C., Maddirevula, S., Cheng, Y.H., Phelps, I.G., et al.; University of Washington Center for Mendelian Genomics (2017). Mutations in *ARMC9*, which encodes a basal body protein, cause Joubert syndrome in humans and ciliopathy phenotypes in zebrafish. Am. J. Hum. Genet. *101*, 23–36.
8. Sang, L., Miller, J.J., Corbit, K.C., Giles, R.H., Brauer, M.J., Otto, E.A., Baye, L.M., Wen, X., Scales, S.J., Kwong, M., et al. (2011). Mapping the NPHP-JBTS-MKS protein network reveals ciliopathy disease genes and pathways. Cell *145*, 513–528.
9. Wheway, G., Schmidts, M., Mans, D.A., Szymanska, K., Nguyen, T.T., Racher, H., Phelps, I.G., Toedt, G., Kennedy, J., Wunderlich, K.A., et al.; UK10K Consortium; and University of Washington Center for Mendelian Genomics (2015). An siRNA-based functional genomics screen for the identification of regulators of ciliogenesis and ciliopathy genes. Nat. Cell Biol. *17*, 1074–1087.
10. Shaheen, R., Szymanska, K., Basu, B., Patel, N., Ewida, N., Faqeih, E., Al Hashem, A., Derar, N., Alsharif, H., Aldahmesh, M.A., et al.; Ciliopathy WorkingGroup (2016). Characterizing the morbid genome of ciliopathies. Genome Biol. *17*, 242.
11. Otto, E.A., Hurd, T.W., Airik, R., Chaki, M., Zhou, W., Stoetzel, C., Patil, S.B., Levy, S., Ghosh, A.K., Murga-Zamalloa, C.A., et al. (2010). Candidate exome capture identifies mutation of *SDCCAG8* as the cause of a retinal-renal ciliopathy. Nat. Genet. *42*, 840–850.
12. Chaki, M., Airik, R., Ghosh, A.K., Giles, R.H., Chen, R., Slaats, G.G., Wang, H., Hurd, T.W., Zhou, W., Cluckey, A., et al. (2012). Exome capture reveals *ZNF423* and *CEP164* mutations, linking renal ciliopathies to DNA damage response signaling. Cell *150*, 533–548.
13. Hanke-Gogokhia, C., Wu, Z., Gerstner, C.D., Frederick, J.M., Zhang, H., and Baehr, W. (2016). Arf-like protein 3 (ARL3) regulates protein trafficking and ciliogenesis in mouse photoreceptors. J. Biol. Chem. *291*, 7142–7155.
14. Zhang, Q., Hu, J., and Ling, K. (2013). Molecular views of Arf-like small GTPases in cilia and ciliopathies. Exp. Cell Res. *319*, 2316–2322.
15. Ismail, S.A., Chen, Y.X., Miertzschke, M., Vetter, I.R., Koerner, C., and Wittinghofer, A. (2012). Structural basis for Arl3-specific release of myristoylated ciliary cargo from UNC119. EMBO J. *31*, 4085–4094.
16. Ismail, S.A., Chen, Y.X., Rusinova, A., Chandra, A., Bierbaum, M., Gremer, L., Triola, G., Waldmann, H., Bastiaens, P.I., and Wittinghofer, A. (2011). Arl2-GTP and Arl3-GTP regulate a GDI-like transport system for farnesylated cargo. Nat. Chem. Biol. *7*, 942–949.
17. Veltel, S., Gasper, R., Eisenacher, E., and Wittinghofer, A. (2008). The retinitis pigmentosa 2 gene product is a GTPase-activating protein for Arf-like 3. Nat. Struct. Mol. Biol. *15*, 373–380.
18. Grayson, C., Bartolini, F., Chapple, J.P., Willison, K.R., Bhamidipati, A., Lewis, S.A., Luthert, P.J., Hardcastle, A.J., Cowan, N.J., and Cheetham, M.E. (2002). Localization in the human retina of the X-linked retinitis pigmentosa protein RP2, its homologue cofactor C and the RP2 interacting protein Arl3. Hum. Mol. Genet. *11*, 3065–3074.
19. Gotthardt, K., Lokaj, M., Koerner, C., Falk, N., Gießl, A., and Wittinghofer, A. (2015). A G-protein activation cascade from Arl13B to Arl3 and implications for ciliary targeting of lipidated proteins. eLife *4*, e11859.
20. Fansa, E.K., Kösling, S.K., Zent, E., Wittinghofer, A., and Ismail, S. (2016). PDE6δ-mediated sorting of INPP5E into the cilium is determined by cargo-carrier affinity. Nat. Commun. *7*, 11366.
21. Lokaj, M., Kösling, S.K., Koerner, C., Lange, S.M., van Beersum, S.E., van Reeuwijk, J., Roepman, R., Horn, N., Ueffing, M., Boldt, K., and Wittinghofer, A. (2015). The interaction of CCDC104/BARTL1 with Arl3 and implications for ciliary function. Structure *23*, 2122–2132.
22. Jaiswal, M., Fansa, E.K., Kösling, S.K., Mejuch, T., Waldmann, H., and Wittinghofer, A. (2016). Novel biochemical and structural insights into the interaction of myristoylated cargo with Unc119 protein and their release by Arl2/3. J. Biol. Chem. *291*, 20766–20778.
23. Haycraft, C.J., Banizs, B., Aydin-Son, Y., Zhang, Q., Michaud, E.J., and Yoder, B.K. (2005). Gli2 and Gli3 localize to cilia and require the intraflagellar transport protein polaris for processing and function. PLoS Genet. *1*, e53.
24. Cuvillier, A., Redon, F., Antoine, J.C., Chardin, P., DeVos, T., and Merlin, G. (2000). LdARL-3A, a Leishmania promastigote-specific ADP-ribosylation factor-like protein, is essential for flagellum integrity. J. Cell Sci. *113*, 2065–2074.
25. Schrick, J.J., Vogel, P., Abuin, A., Hampton, B., and Rice, D.S. (2006). ADP-ribosylation factor-like 3 is involved in kidney and photoreceptor development. Am. J. Pathol. *168*, 1288–1298.
26. Hartill, V., Szymanska, K., Sharif, S.M., Wheway, G., and Johnson, C.A. (2017). Meckel-Gruber syndrome: An update on diagnosis, clinical management, and research advances. Front Pediatr. *5*, 244.
27. Strom, S.P., Clark, M.J., Martinez, A., Garcia, S., Abelazeem, A.A., Matynia, A., Parikh, S., Sullivan, L.S., Bowne, S.J., Daiger, S.P., and Gorin, M.B. (2016). *De novo* occurrence of a variant in *ARL3* and apparent autosomal dominant transmission of retinitis pigmentosa. PLoS ONE *11*, e0150944.
28. Pierce, E.A., Quinn, T., Meehan, T., McGee, T.L., Berson, E.L., and Dryja, T.P. (1999). Mutations in a gene encoding a new oxygen-regulated photoreceptor protein cause dominant retinitis pigmentosa. Nat. Genet. *22*, 248–254.
29. Khaliq, S., Abid, A., Ismail, M., Hameed, A., Mohyuddin, A., Lall, P., Aziz, A., Anwar, K., and Mehdi, S.Q. (2005). Novel association of *RP1* gene mutations with autosomal recessive retinitis pigmentosa. J. Med. Genet. *42*, 436–438.

30. Gerber, S., Alzayady, K.J., Burglen, L., Brémond-Gignac, D., Marchesin, V., Roche, O., Rio, M., Funalot, B., Calmon, R., Durr, A., et al. (2016). Recessive and dominant de novo *ITPR1* mutations cause Gillespie syndrome. Am. J. Hum. Genet. *98*, 971–980.
31. Monies, D., Maddirevula, S., Kurdi, W., Alanazy, M.H., Alkhalidi, H., Al-Owain, M., Sulaiman, R.A., Faqeih, E., Goljan, E., Ibrahim, N., et al. (2017). Autozygosity reveals recessive mutations and novel mechanisms in dominant genes: implications in variant interpretation. Genet. Med. *19*, 1144–1150.
32. Cantagrel, V., Silhavy, J.L., Bielas, S.L., Swistun, D., Marsh, S.E., Bertrand, J.Y., Audollent, S., Attié-Bitach, T., Holden, K.R., Dobyns, W.B., et al.; International Joubert Syndrome Related Disorders Study Group (2008). Mutations in the cilia gene *ARL13B* lead to the classical form of Joubert syndrome. Am. J. Hum. Genet. *83*, 170–179.
33. Thomas, S., Wright, K.J., Le Corre, S., Micalizzi, A., Romani, M., Abhyankar, A., Saada, J., Perrault, I., Amiel, J., Litzler, J., et al. (2014). A homozygous *PDE6D* mutation in Joubert syndrome impairs targeting of farnesylated INPP5E protein to the primary cilium. Hum. Mutat. *35*, 137–146.
34. Bielas, S.L., Silhavy, J.L., Brancati, F., Kisseleva, M.V., Al-Gazali, L., Sztriha, L., Bayoumi, R.A., Zaki, M.S., Abdel-Aleem, A., Rosti, R.O., et al. (2009). Mutations in *INPP5E*, encoding inositol polyphosphate-5-phosphatase E, link phosphatidyl inositol signaling to the ciliopathies. Nat. Genet. *41*, 1032–1036.
35. Lewis, K.E., Drossopoulou, G., Paton, I.R., Morrice, D.R., Robertson, K.E., Burt, D.W., Ingham, P.W., and Tickle, C. (1999). Expression of ptc and gli genes in talpid3 suggests bifurcation in Shh pathway. Development *126*, 2397–2407.
36. Stephen, L.A., Tawamie, H., Davis, G.M., Tebbe, L., Nürnberg, P., Nürnberg, G., Thiele, H., Thoenes, M., Boltshauser, E., Uebe, S., et al. (2015). TALPID3 controls centrosome and cell polarity and the human ortholog *KIAA0586* is mutated in Joubert syndrome (*JBTS23*). eLife *4*, e08077.
37. Olbrich, H., Fliegauf, M., Hoefele, J., Kispert, A., Otto, E., Volz, A., Wolf, M.T., Sasmaz, G., Trauer, U., Reinhardt, R., et al. (2003). Mutations in a novel gene, *NPHP3*, cause adolescent nephronophthisis, tapeto-retinal degeneration and hepatic fibrosis. Nat. Genet. *34*, 455–459.
38. Tory, K., Rousset-Rouvière, C., Gubler, M.C., Morinière, V., Pawtowski, A., Becker, C., Guyot, C., Gié, S., Frishberg, Y., Nivet, H., et al. (2009). Mutations of *NPHP2* and *NPHP3* in infantile nephronophthisis. Kidney Int. *75*, 839–847.
39. Bergmann, C., Fliegauf, M., Brüchle, N.O., Frank, V., Olbrich, H., Kirschner, J., Schermer, B., Schmedding, I., Kispert, A., Kränzlin, B., et al. (2008). Loss of nephrocystin-3 function can cause embryonic lethality, Meckel-Gruber-like syndrome, situs inversus, and renal-hepatic-pancreatic dysplasia. Am. J. Hum. Genet. *82*, 959–970.
40. Humbert, M.C., Weihbrecht, K., Searby, C.C., Li, Y., Pope, R.M., Sheffield, V.C., and Seo, S. (2012). ARL13B, PDE6D, and CEP164 form a functional network for INPP5E ciliary targeting. Proc. Natl. Acad. Sci. USA *109*, 19691–19696.
41. Zimmermann, G., Papke, B., Ismail, S., Vartak, N., Chandra, A., Hoffmann, M., Hahn, S.A., Triola, G., Wittinghofer, A., Bastiaens, P.I., and Waldmann, H. (2013). Small molecule inhibition of the KRAS-PDEδ interaction impairs oncogenic KRAS signalling. Nature *497*, 638–642.
42. Mejuch, T., Garivet, G., Hofer, W., Kaiser, N., Fansa, E.K., Ehrt, C., Koch, O., Baumann, M., Ziegler, S., Wittinghofer, A., and Waldmann, H. (2017). Small-molecule inhibition of the UNC119-cargo interaction. Angew. Chem. Int. Ed. Engl. *56*, 6181–6186.
43. Dixon-Salazar, T., Silhavy, J.L., Marsh, S.E., Louie, C.M., Scott, L.C., Gururaj, A., Al-Gazali, L., Al-Tawari, A.A., Kayserili, H., Sztriha, L., and Gleeson, J.G. (2004). Mutations in the *AHI1* gene, encoding jouberin, cause Joubert syndrome with cortical polymicrogyria. Am. J. Hum. Genet. *75*, 979–987.
44. Ferland, R.J., Eyaid, W., Collura, R.V., Tully, L.D., Hill, R.S., Al-Nouri, D., Al-Rumayyan, A., Topcu, M., Gascon, G., Bodell, A., et al. (2004). Abnormal cerebellar development and axonal decussation due to mutations in *AHI1* in Joubert syndrome. Nat. Genet. *36*, 1008–1013.

The American Journal of Human Genetics, Volume *103*

Supplemental Data

ARL3 Mutations Cause Joubert Syndrome by Disrupting Ciliary Protein Composition

Sumaya Alkanderi, Elisa Molinari, Ranad Shaheen, Yasmin Elmaghloob, Louise A. Stephen, Veronica Sammut, Simon A. Ramsbottom, Shalabh Srivastava, George Cairns, Noel Edwards, Sarah J. Rice, Nour Ewida, Amal Alhashem, Kathryn White, Colin G. Miles, David H. Steel, Fowzan S. Alkuraya, Shehab Ismail, and John A. Sayer

Supplemental Figures

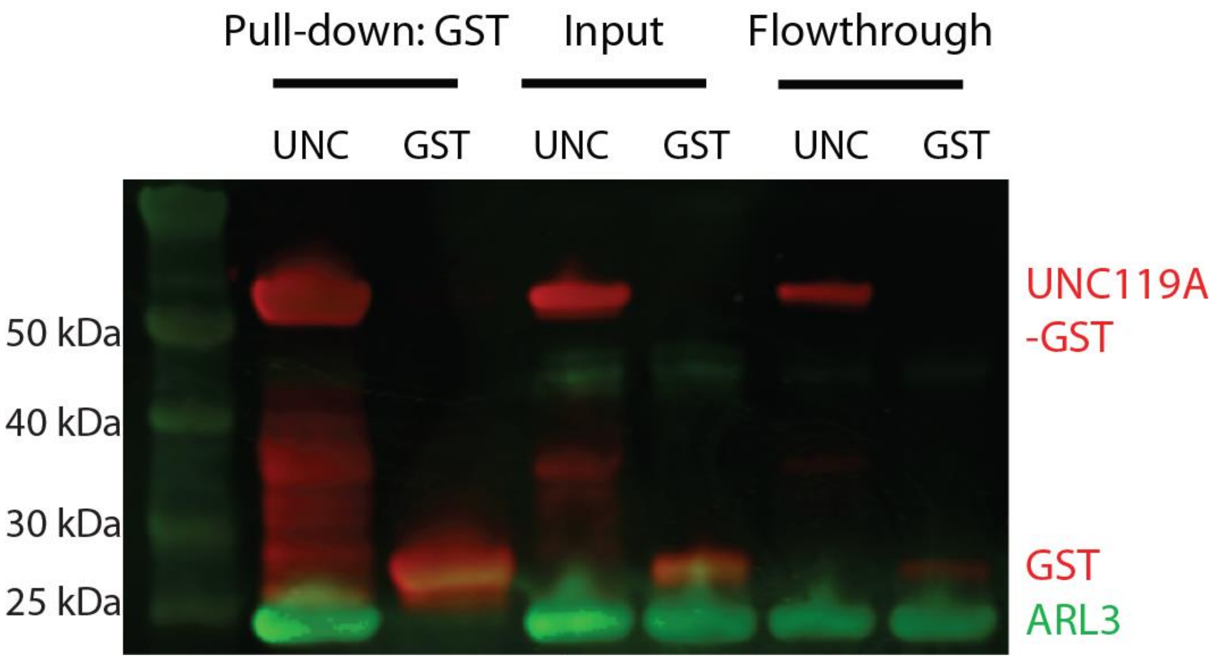

Figure S1. ARL3 specifically interacts with UNC119A

30 µg of full-length UNC119A-GST (labelled UNC) or GST alone (labelled GST) were used to pull down 60 µg of murine ARL3^{WT} that was loaded with the GTP analogue GppNHp. Proteins were detected on immunoblots using anti-GST (red) and anti-His (green) antibodies.

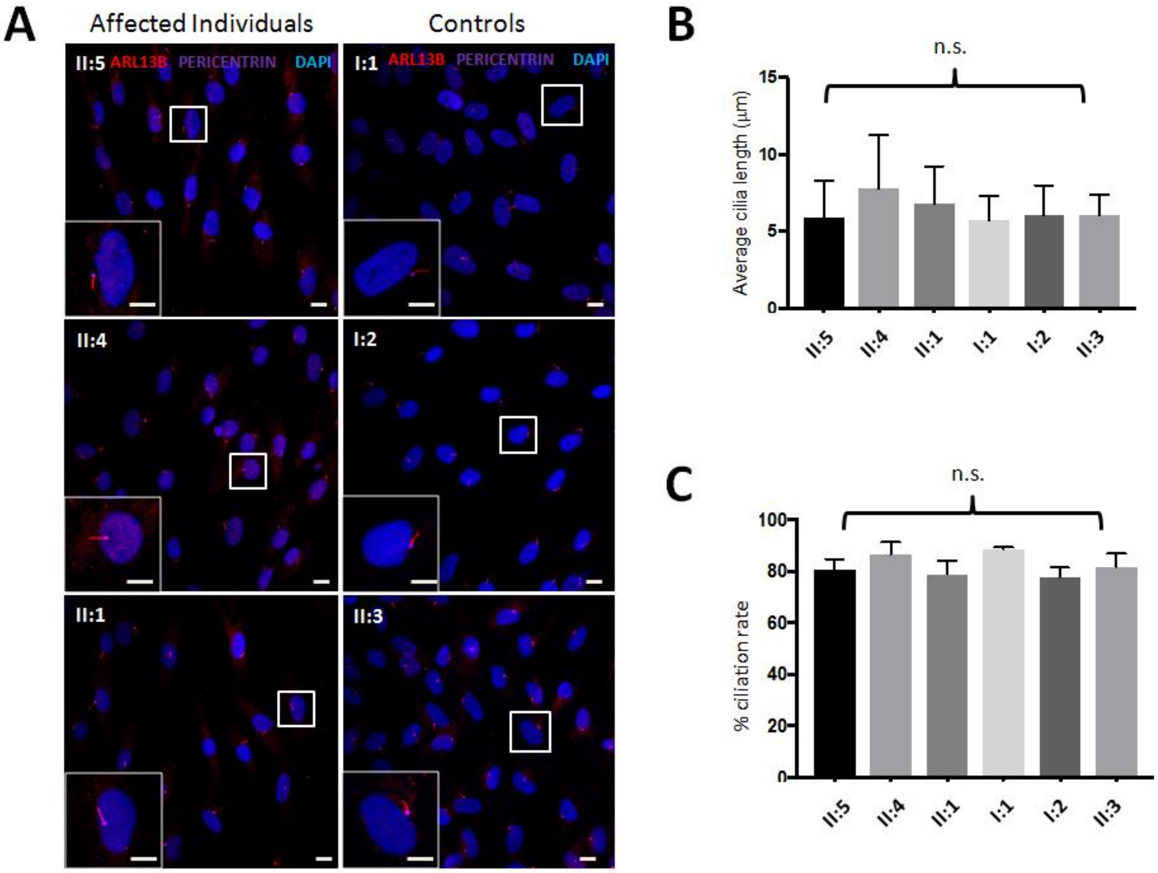

Figure S2. Analysis of cilia length using immunofluorescence in affected individuals and unaffected relative fibroblasts

(A) Fibroblasts from affected individuals in family 2 (II:5, II:4, II:1) and controls (I:1, I;2, II:3) were imaged using immunofluorescence microscopy following staining with anti-ARL13B (red) to identify ciliary membrane, anti-PERICENTRIN (magenta) to identify the ciliary base and DAPI (blue) to identify the cell nucleus. Scale bar = 10 µm.

(B) Quantification of cilia length from affected and control fibroblasts (n.s. not significant, ANOVA).

(C) Percentage ciliation rate in affected and control fibroblasts (n.s. not significant, ANOVA).

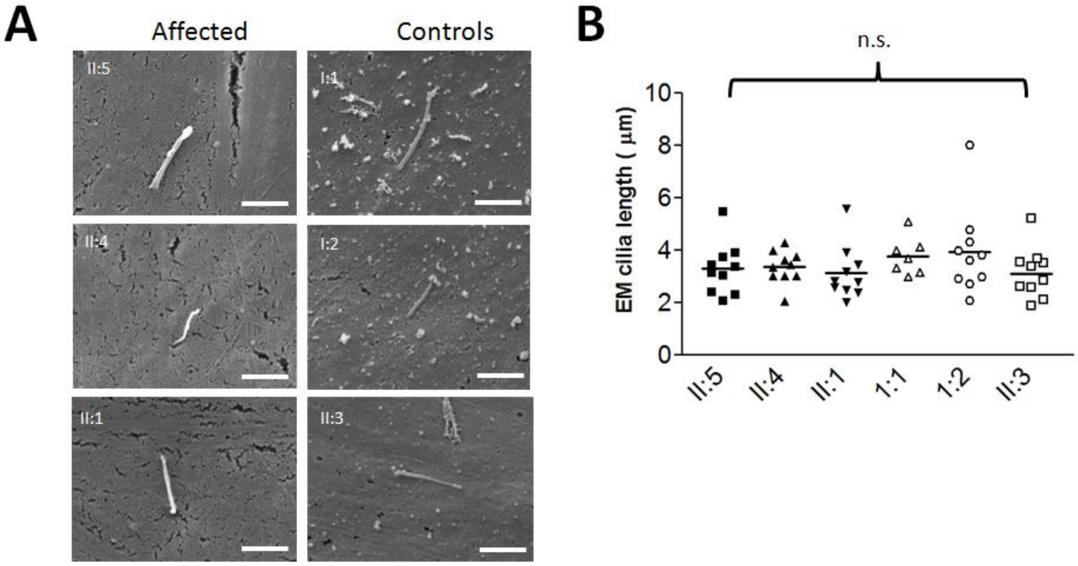

Figure S3. Scanning electron microscopy and quantification of cilia length in *ARL3* mutant and control fibroblasts

(A) Fibroblasts from affected individuals in family 2 (II:5, II:4, II:1) and controls (I:1, I;2, II:3) seen under scanning EM reveal normal appearances of primary cilia (Scale bar 2 µm).

(B) Dot plot to show quantification of EM cilia length, bars represent mean (n.s. not significant, ANOVA).

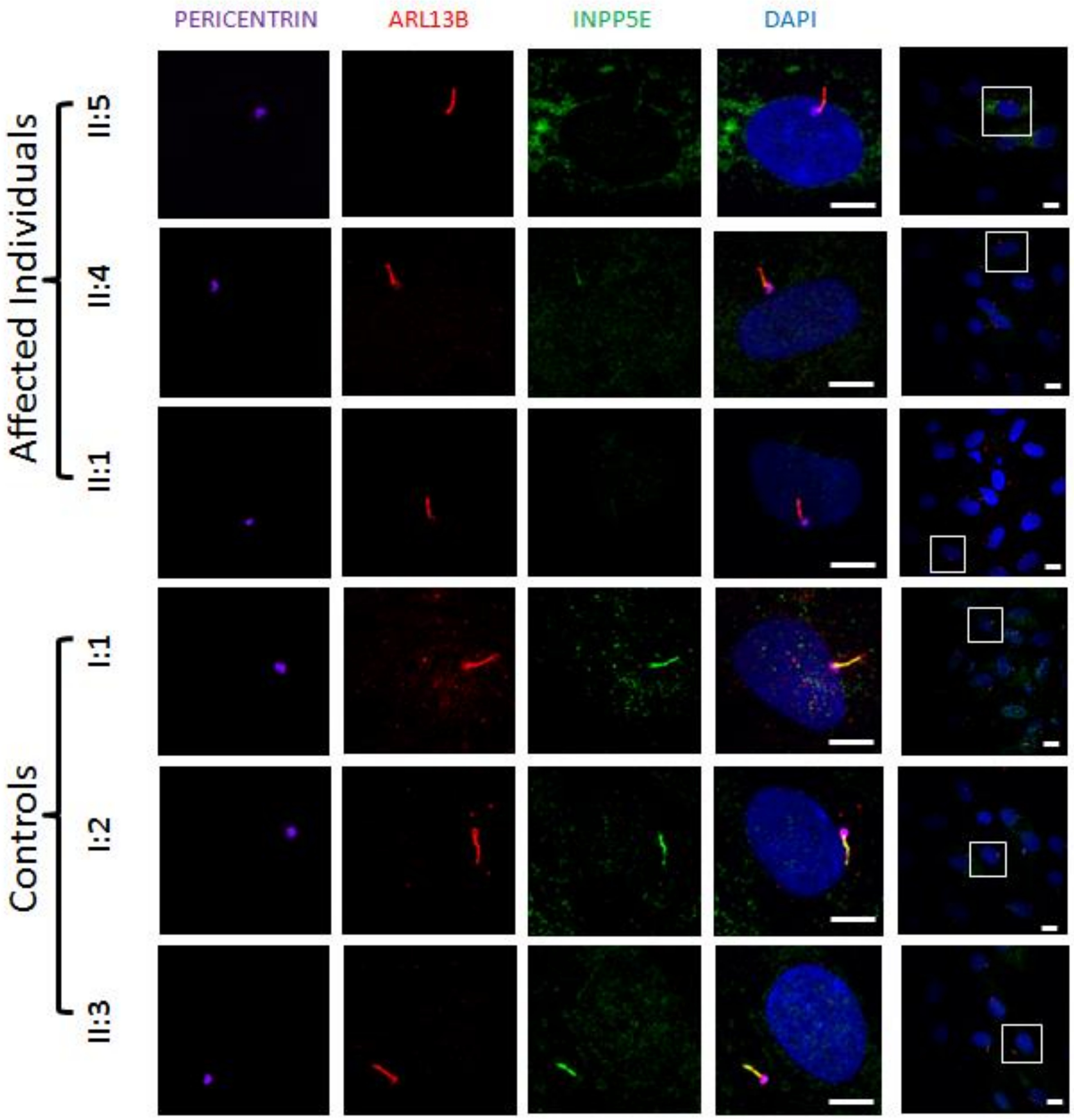

Figure S4. Analysis of INPP5E content in fibroblast cilia

Fibroblasts from affected individuals in family 2 (II:5, II:4, II:1) and controls (I:1, I:2, II:3) were fixed and stained with antibodies directed towards PERICENTRIN (magenta), ARL13B (red), INPP5E (green) and DAPI (blue) as a nuclear marker. Representative individual images are shown for PERICENTRIN, ARL13B and INPP5E as well as overlay images at high and low power. Scale bar = 10 µm.

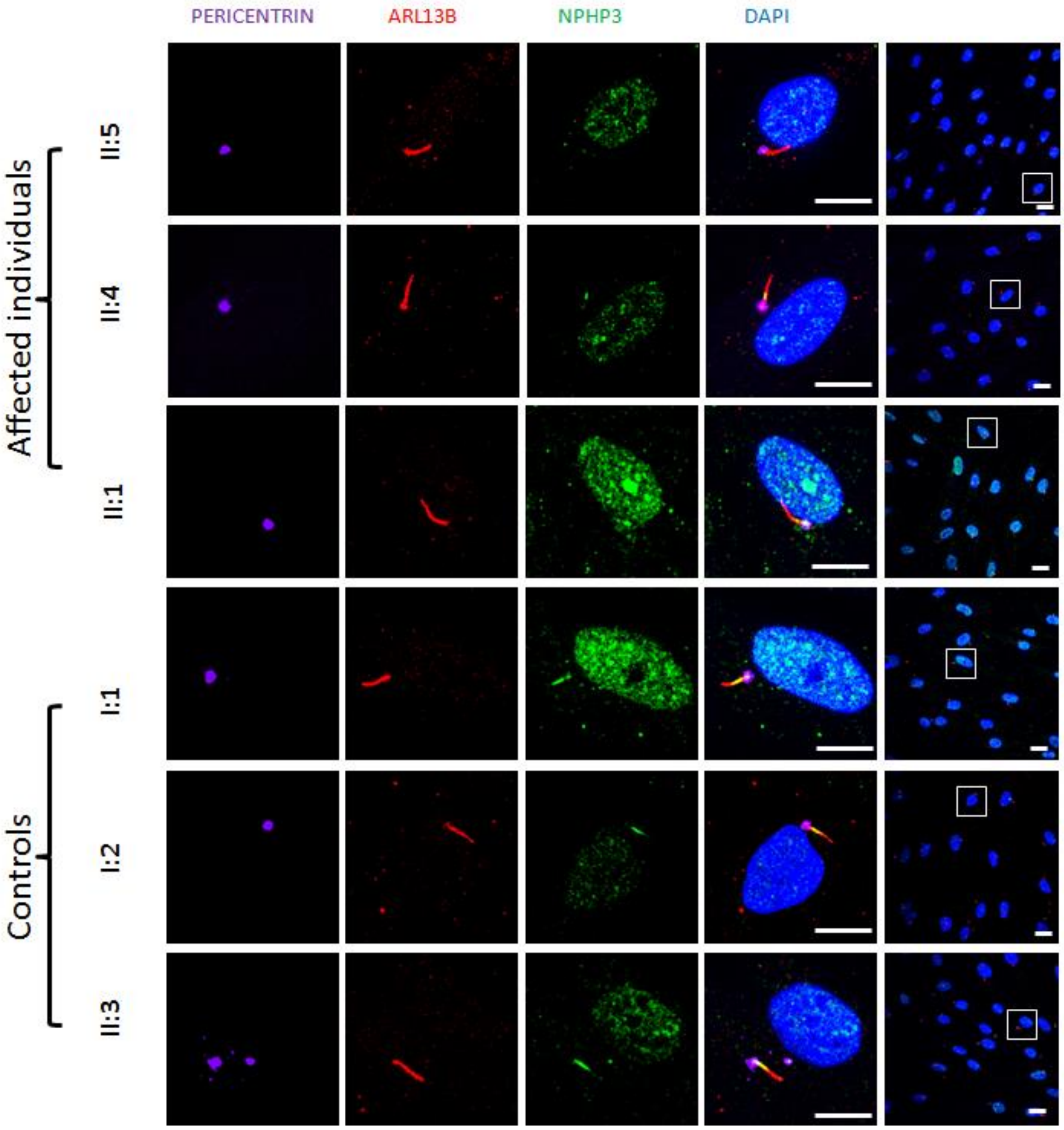

Figure S5. Analysis of NPHP3 content in fibroblast cilia

Fibroblasts from affected individuals in family 2 (II:5, II:4, II:1) and controls (I:1, I:2, II:3) were fixed and stained with antibodies directed towards PERICENTRIN (magenta), ARL13B (red), NPHP3 (green) and DAPI (blue) as a nuclear marker. Representative individual images are shown for PERICENTRIN, ARL13B and NPHP3 as well as overlay images at high and low power. Scale bar = 10 µm. Note, intracellular NPHP3 staining (green) is variable, does not correspond to genotype and may be non-specific.

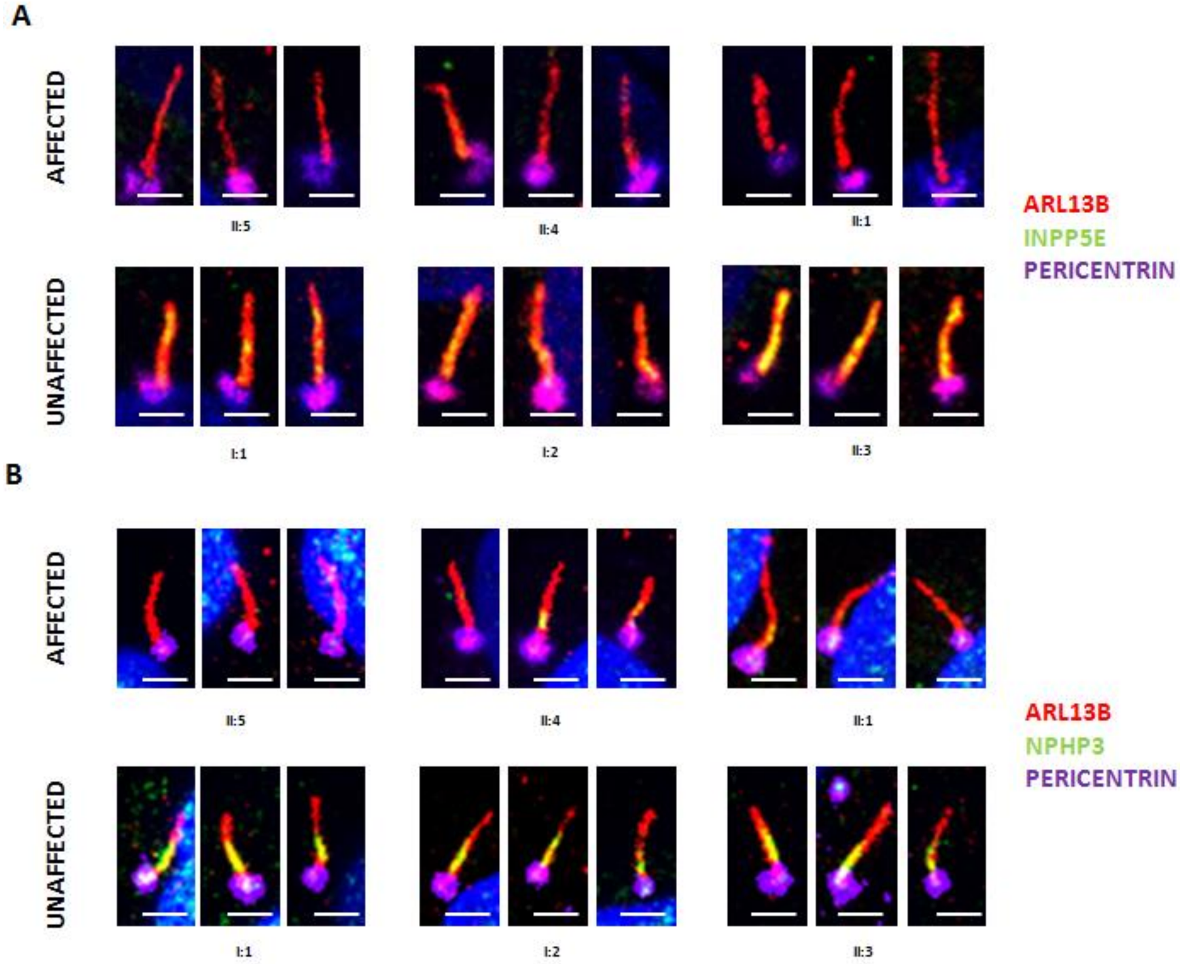

Figure S6. INPP5E and NPHP3 content in fibroblast cilia

Zoomed overlay images of fibroblast primary cilia from affected individuals in family 2 (II:5, II:4, II:1) and controls (I:1, I:2, II:3) were fixed and stained with antibodies directed towards PERICENTRIN (magenta), ARL13B (red), and (A) INPP5E (green) and (B) NPHP3 (green) and DAPI (blue) as a nuclear marker.

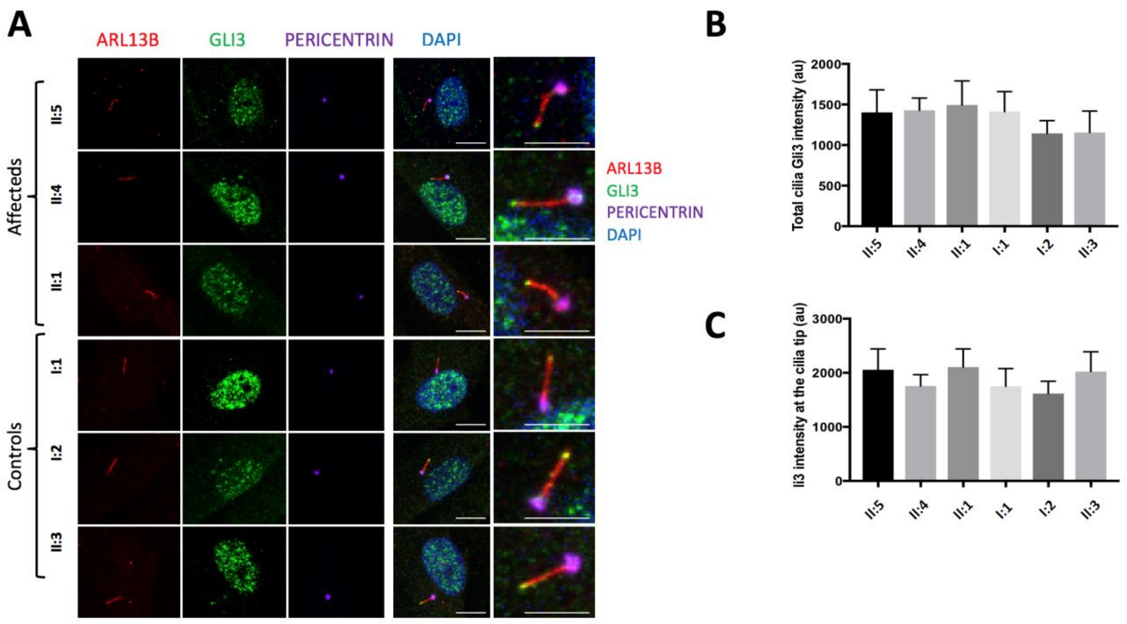

Figure S7. Lack of disruption of GLI3 localisation in cilia axoneme and tip in ARL3 mutant fibroblasts

(A) Fibroblasts from affected individuals in family 2 (II:5, II:4, II:1) and controls (I:1, I:2, II:3) were treated with 100 nM SAG then fixed and stained with antibodies directed towards ARL13B (red), GLI3 (green) PERICENTRIN (magenta) and DAPI (blue) as a nuclear marker. Representative single channel images are shown for ARL13B, GLI3 and PERICENTRIN as well as overlay images at low power (scale bar = 10 µm) and zoomed (scale bar = 5 µm) .

(B) Quantification of ciliary localisation of GLI3 is shown (n >35 for each group, n.s. not significant, ANOVA).

(C) Quantification of ciliary tip localisation of GLI3 is shown (n >35 for each group, n.s. not significant, ANOVA).

Supplementary tables

	Family 1	Family 2	
Affected member	II:5	II:4	II:5
Number of variants	52809	145724	144048
Homozygous coding / splicing < 0.1% in 1000 Genome database, gnomAD, ExAC and in-house ethnically matched exomes	16	42	31
Within the homozygous regions	11	8	15
Predicted to be pathogenic	2	5	3
Biological Context (Known JBTS genes)	0	0	0
Within the shared ROH (3 affected)	1	1	1

Table S1. Filtering criteria to the WES files of the three affected members from the two families included in this study.

Family 1 II:5	Reason for inclusion /exclusion
**Chr10. *ARL3*:**<br>NM_004311.3: c.445C>T, p.(Arg149Cys)	Include: candidate ciliopathy gene
**Chr8. *MTMR7*:**<br>NM_004686.4:c.547C>T, p.(Arg183Trp)	Exclude: It encodes a myotubularin related protein. Mutation in *MTMR1*, which encode a similar member of the myotubularin related family of proteins cause Charcot-Marie-Tooth neuropathies.
Family 2 II:4	
**Chr10. *ARL3***<br>NM_004311.3: c.446G>A, p.(Arg149His)	Include: Candidate ciliopathy gene
**Chr11. *PKP3***<br>NM_007183.3: c.649C.T, p.(Arg217Cys)	Exclude: encodes a desmosomal plaque protein. Variant not found in sibling or Family 1
**Chr11. *OR52N4***<br>NM_001005175.3: c.656C>T, p. (Ser219Phe)	Exclude: Olfactory Receptor family member. Variant not found in sibling or Family 1
**Chr13. *FAM124A***<br>NM_145019.3: c.1717G>T, p.(Gly573Trp)	Exclude: Family With Sequence Similarity 124 Member A. Variant not found in sibling or Family 1
**Chr17. *SLC5A10***<br>NM.001042450.2, c.1412G>A, p.(Gly435Glu)	Exclude: Sodium/glucose transporter, expression limited to kidney. Variant not found in sibling or Family 1
Family 2 II:5	
**Chr7. *CTAGE9***<br>NM_001008747.2, c.306G>C, p.(Glu102Asp)	Exclude: cutaneous T-cell lymphoma-associated antigen 9. Variant not found in sibling or Family 1
**Chr10. *ARL3***<br>NM_004311.3: c.446G>A, p.(Arg149His)	Include: Candidate ciliopathy gene
**Chr12. *DPY19L2***<br>NM_173812.4, c.1660A>G, p.(Met554Cys)	Exclude: known phenotype of spermatogenic failure (MIM 613893). Variant not found in sibling or Family 1

Table S2. Predicted Pathogenic Homozygous Variants Found

Nucleotide change (Ref sequence NM_004311.3)	Amino acid change	Mutation Taster	PolyPhen 2	SIFT	CADD Score	gnomAD / ExAC allele frequency
c.445C>T	p.(Arg149 Cys)	Disease Causing	Probably damaging (0.912)	Deleterious (0)	35	0.0000325 / 0.00003298
c.446G>A	p.(Arg149 His)	Disease Causing	Possibly damaging (0.758)	Deleterious (0.02)	34	0.000004063/ 0.00000824

Table S3. ***In silico*** **analysis of the** ***ARL3*** **mutations**

CADD Score, Combined Annotation Dependent Depletion, score >20 indicates the 1% most deleterious amino acid substitution

Supplemental materials and methods

Ethics and informed consent

All affected individuals and their relatives consented to this study. For Saudi Arabian individuals, ethical approval and informed consent was obtained under an IRB-approved research protocol (KFSRHC RAC #2080006)). For UK individuals, ethical approval was obtained from the UK National Research Ethics Service (NRES) Committee Northern and Yorkshire (09/H0903/36) and NRES Committee North East – Newcastle and North Tyneside 1 (08/H0906/28+5). Following informed and written consent, blood samples were obtained from affected individuals and their relatives and healthy gender and age-matched controls. Skin fibroblasts were obtained from affected individuals and their relatives following informed written consent and stored in Newcastle MRC Centre Biobank for Neuromuscular and Rare Diseases.

Whole exome sequencing and analysis

Genomic DNA (2µg) from two affected siblings and their parents was subjected to whole exome paired-end sequencing analysis. Ingenuity Variant Analysis web-based application and HomozygosityMapper (http://www.homozygositymapper.org/) were used for data analysis. The variants of interest were further tested for likely pathogenicity using *in silico* prediction tools. The variants were confirmed using Sanger sequencing in all available family members to confirm segregation with disease status. Gene variants have been submitted to www.LOVD.nl/ARL3 (patient IDs 00179519, 00179520, 00179521, 00179522).

Homology modelling

Human ARL3 (UniProt accession code P36405) and ARL13B (UniProt accession code Q3SXY8) were modelled on the crystal structure of the Arl3-Arl13B complex from *Chlamydomonas reinhardtii* (PDB accession code 5DI3) using HHPred [1] and Modeller [2] software. Structures were visualised and figures prepared using PyMOL (http://www.pymol.org/).

Fibroblast culture, immunofluorescence imaging and quantification

Fibroblasts were isolated from skin biopsies, cultured in DMEM, 10% foetal bovine serum (FBS) and 1% Pen/Strep and incubated in 37 °C. For cilia imaging, cells were seeded on coverslips and grown to 100% confluency then starved for 48 h (in FBS free media). For GLI3 ciliary trafficking studies cells were treated with 100 nM Smoothened Agonist (SAG) after 24 h of starvation then incubated for another 2 h in FBS

free media. Cells were fixed with 100% ice-cold methanol for 15 min, then washed three times with phosphate-buffered saline (PBS). Fixed cells were incubated in PBS with 10% bovine serum albumin (BSA), as a blocking agent. For indirect immunostaining of primary cilia, primary antibodies were diluted in blocking solution. Cells were incubated in INPP5E 1:100 (Proteintech 177197-1-AP), GLI3 1:200 (Abcam ab69838) and PERICENTRIN 1:1000 (Abcam ab4448) at room temperature for 1 h or in NPHP3 1:100 (Proteintech 22026-1-AP) at 4 °C overnight. For secondary staining, cells were incubated at room temperature for 1 h with Alexa Fluor conjugated secondary antibodies diluted in blocking solution. For direct immunostaining of primary cilia, Zenon Alexa Fluor 555 Rabbit IgG labelling kit (Thermo Fisher Z25305) was used, with primary antibody ARL13B 1:400 (Proteintech 17711-1AP) diluted 1:3 in PBS with 5ul of labelling mix. Coverslips were mounted on glass slides using mounting medium with DAPI (Vectashield). Images were obtained with confocal microscopy (NIKON A1), using z-stacks and identical laser intensity as well as camera settings and imported as TIFF files. Image analysis was performed using Fiji (ImageJ) software.

For quantification of percentage of cilia, five 60X fields were analysed (nuclei vs cilia identified) then a mean score was calculated. The segmented line tool within Fiji (ImageJ) was used to measure cilia length from base of axoneme (labelled with PERICENTRIN) to the distal cilia tip. For antibody signal intensity quantification experiments, 4-5 60X fields were analysed. The freehand tool was used to mark cilia borders using ARL13B signal and a region of interest (ROI) was constructed to measure the mean signal intensity of INPP5E, NPHP3 or GLI3. Then ROI was duplicated and dragged to nearby area to correct for local background fluorescence intensity by simple subtraction. For cilia tip GLI3 signal intensity, freehand tool was used to mark cilia tip borders, mean signal intensity of labelled area (0.5-1.0 micron) was measured then corrected for local background intensity.

Scanning electron microscopy imaging

For scanning electron microscopy, cells were fixed overnight in 2% glutaraldehyde in 0.1 M Sorenson's phosphate buffer, dehydrated through a graded series of ethanol and then critical-point dried (Baltec dryer). Samples were coated with 10nm of gold (olaron coating unit) and viewed on a Tescan Vega LMU SEM operated at 8–10 kV. Images were captured and measured in a blinded fashion.

Protein expression and purification

Chlamydomonas reinhardtii and murine full-length ARL3, *Chlamydomonas reinhardtii* ARL13B 18-278, and human full-length UNC119A were expressed and purified as previously described [3]. Human ARL13B 18-278 was cloned into pET20b with an N-terminal 12×His-tag and purified using the same protocol as ARL3 with the addition of 10% glycerol to the purification buffers and storing the protein in 5% glycerol. p.Arg149His mutations in *ARL3* and p.Glu86Arg mutation in *ARL13B* were introduced using the Q5 site-directed mutagenesis kit (NEB).

Guanine nucleotide exchange assay

Murine ARL3 used for GST pull-downs was exchanged with the non-hydrolysable analogue of GTP, GppNHp. 300 µM ARL3 was incubated with 2 mM GppNHp and 0.14 U/µL Alkaline Phosphatase (Roche) overnight at 15°C. Excess nucleotides were removed by size exclusion chromatography on a Superdex S200 Increase column (GE Healthcare). GppNHp-loaded ARL3 was quantified using HPLC analysis and C18 columns (UltiMate3000, Thermo Fisher Scientific). For the GEF assay, nucleotide exchange of murine and *Chlamydomonas reinhardtii* ARL3 proteins with mantGDP was carried out by incubating 100 µM ARL3 protein with 200 µM mantGDP for 2 h at room temperature in the presence of 50 mM EDTA. For nucleotide exchange to GppNHp on human and *Chlamydomonas reinhardtii* ARL13B, 100 µM ARL13B was incubated with 500 µM GppNHp and 50 mM EDTA, also for 2 h at room pressure. All reactions were carried out in 20 mM tris pH 7.5, 150 mM NaCl, 5 mM $MgCl_2$ and 2 mM DTT and were stopped by adding 100 mM $MgCl_2$. Unbound nucleotides were removed by using a PD10 desalting column (GE Healthcare), and the proteins were concentrated using Amicon Ultra 0.5 mL units (Merck Millipore) with a molecular weight cut-off of 3 kDa.

All reactions were carried out in 20 mM Tris pH 7.5, 150 mM NaCl, 5 mM $MgCl_2$ and 2 mM DTT at room temperature. Fluorescence polarisation was measured at an excitation of 366 nm and emission of 450 nm. 0.5 µM of *Cr*ARL3•mantGDP was measured for 100 s, after which 10 µM GppNHp was added and measured for an additional 100 s. Finally, 5 µM of *Cr*ARL13B•GppNHp was added and changes in fluorescence polarisation were recorded for 300 s. The same was repeated for *Hs*ARL13B, but starting with 1 µM of murine ARL3•mantGDP to which 400 µM GppNHp was added followed by 5 µM *Hs*ARL13B.

GST pull-downs

30 µg of GST-tagged full-length UNC119A and 60 µg of GppNHp-loaded murine ARL3 WT and p.Arg149His were incubated in 20 mM Tris pH 7.5, 150 mM NaCl, 5 mM $MgCl_2$ and 2 mM DTT for 15 minutes at room temperature before adding to Glutathione sepharose 4 FF beads (GE Healthcare) and incubating for a further 20 minutes. Beads were washed 5 times in reaction buffer before eluting with reaction buffer containing 20 mM Glutathione.

Protein Constructs

The following protein constructs were used for protein-protein interaction studies:

Chlamydomonas reinhardtii $ARL3^{WT}$ (C-terminal 6xHis-tag)

MGLLSLIRGL KKKEGEARIL VLGLDNAGKT TILKALSEED ITTITPTQGF NIKSLSRDGF NLKIWDIGGQ KSIRPYWRNY FDQTDALIYV IDSADSKRLS ESEFELTELL QEEKMTGVPL LVFANKQDLV GALAADEIAS TLDLTSIRDR PWQIQACSAK QGTGLKEGME WMMKQVK

Chlamydomonas reinhardtii $ARL3^{R148H}$ (C-terminal 6xHis-tag)

MGLLSLIRGL KKKEGEARIL VLGLDNAGKT TILKALSEED ITTITPTQGF NIKSLSRDGF NLKIWDIGGQ KSIRPYWRNY FDQTDALIYV IDSADSKRLS ESEFELTELL QEEKMTGVPL LVFANKQDLV GALAADEIAS TLDLTSIHDR PWQIQACSAK QGTGLKEGME WMMKQVK

Chlamydomonas reinhardtii ARL13B 18-278 (N-terminal GST-tag)

KITIALLGLD NAGKTTLLNS IQGEVDRDTT PTFGFNSTTL NEGKYKIEVF DLGGGKNIRG VWKKYLAEVH AIVYVVDAAD PGRFEESKMT MAEVLENQFM RDKPICIFAN KQDLPTAAPA AEVVKGLGLA TCRNSHNVFP CTAKMPAGQD VDHRLRDGLK WLVGTVDREF GRLDPRVQTE AEEVRQEEAR KKKEREERLR KQREERLRQQ KEEEERAREV EKENELHDGK APSLLAAGGG VVGAAAAGVN GVMVDEQQEL

Mus musculus ARL3^{WT} (C-terminal 6xHis-tag)

MGLLSILRKL KSAPDQEVRI LLLGLDNAGK TTLLKQLASE DISHITPTQG FNIKSVQSQG FKLNVWDIGG QRKIRPYWRS YFENTDILIY VIDSADRKRF EETGQELTEL LEEEKLSCVP VLIFANKQDL LTAAPASEIA EGLNLHTIRD RVWQIQSCSA LTGEGVQDGM NWVCKNVNAK KK

Mus musculus ARL3^{R149H} (C-terminal 6xHis-tag)

MGLLSILRKL KSAPDQEVRI LLLGLDNAGK TTLLKQLASE DISHITPTQG FNIKSVQSQG FKLNVWDIGG QRKIRPYWRS YFENTDILIY VIDSADRKRF EETGQELTEL LEEEKLSCVP VLIFANKQDL LTAAPASEIA EGLNLHTIHD RVWQIQSCSA LTGEGVQDGM NWVCKNVNAK KK

Homo sapiens ARL13B 18-278 (N-terminal 12xHis-tag)

VRKVTLLMVG LDNAGKTATA KGIQGEYPED VAPTVGFSKI NLRQGKFEVT IFDLGGGIRI RGIWKNYYAE SYGVIFVVDS SDEERMEETK EAMSEMLRHP RISGKPILVL ANKQDKEGAL

GEADVIECLS LEKLVNEHKC LCQIEPCSAI SGYGKKIDKS IKKGLYWLLH VIARDFDALN ERIQKETTEQ RALEEQEKQE RAERVRKLRE ERKQNEQEQA ELDGTSGLAE LDPEPTNPFQ PIASVIIENE GKLEREKKNQ

References

1. Soding, J. (2005). Protein homology detection by HMM-HMM comparison. Bioinformatics 21, 951-960.
2. Sali, A., and Blundell, T.L. (1993). Comparative protein modelling by satisfaction of spatial restraints. Journal of molecular biology 234, 779-815.
3. Ismail, S.A., Chen, Y.X., Miertzschke, M., Vetter, I.R., Koerner, C., and Wittinghofer, A. (2012). Structural basis for Arl3-specific release of myristoylated ciliary cargo from UNC119. The EMBO journal 31, 4085-4094.
